# Executive functioning in wild guppies: investigating the impact of a pharmaceutical pollutant

**DOI:** 10.1007/s10071-026-02078-w

**Published:** 2026-06-16

**Authors:** Eleanor R. Moore, Jack L. Manera, Jack A. Brand, Michael G. Bertram, Bob B.M. Wong

**Affiliations:** 1 School of Biological Sciences, Monash University, Melbourne, 3800 Australia; 2 Department of Wildlife, Fish and Environmental Studies, Swedish University of Agricultural Sciences, Umeå, 901 83 Sweden; 3 Institute of Zoology, Zoological Society of London, London, NW1 4RY UK; 4 Department of Zoology, Stockholm University, Stockholm, 114 18 Sweden

**Keywords:** Cognition, Antidepressant, Amitriptyline, Animal behaviour, Cognitive flexibility, Working memory

## Abstract

The executive functions are a suite of cognitive-control processes that enable goal-directed behaviour. Executive functioning is vital in allowing animals to adapt their behaviour to changing environmental conditions, which is a key determinant of the success of individuals, populations, and species under anthropogenic change. Despite this, very little is known about the mechanisms that underpin executive functioning in animals and how it manifests in behaviour. Moreover, whether and how executive functioning is impacted by environmentally realistic levels of chemical pollution is completely unknown, despite neuroactive pollutants, such as pharmaceuticals, having become widespread in ecosystems around the globe. We employed the Free Movement Pattern Y-maze to investigate how executive functioning manifests in decision-making and behaviour in a wild population of guppies (*Poecilia reticulata*), and whether this was disrupted by exposure to two environmentally realistic concentrations (9 ng/L or 309 ng/L) of the neuroactive pharmaceutical pollutant amitriptyline. We found that two of the three domains of executive function—working memory and cognitive flexibility—actively regulated cognition to inform behaviour during maze exploration. However, the role of executive functioning in directing navigation behaviour was not affected by exposure to amitriptyline. Given that neuroactive pharmaceuticals are increasingly detected in waterways, and executive functioning is critical for behaviours that dictate fitness, it is crucial that we better understand whether and how these contaminants affect executive functioning, and what implications this may have for wildlife living in human-impacted ecosystems.

**Supplementary Information:**

The online version contains supplementary material available at https://doi.org/10.1007/s10071-026-02078-w.

## Introduction

Human-induced environmental change presents species with a diverse range of novel challenges that can be drastically different to the conditions under which they evolved. In this regard, behavioural changes are often an animal’s first response to altered environmental conditions (Sih et al. 2011; Wong and Candolin 2014). Individuals that can appropriately modify their behaviour towards new environmental optima are better positioned to cope with these challenges and are therefore more likely to survive and reproduce in a rapidly changing world (Sih 2024; Bertram et al. 2026). Consequently, behavioural flexibility is a key component of individual fitness in human-altered environments, with cascading effects on the survival of populations and species, as well as community and ecosystem dynamics (Sih 2013; Snell-Rood and Steck 2019).

Behavioural flexibility is ultimately a product of executive functioning: cognitive processes that allow adaptive behaviour and decision-making that is goal-directed and informed by both current environmental cues as well as past experiences (Diamond 2013; Logue and Gould 2014). Executive functioning can be divided into three broad domains. First, working memory allows individuals to temporarily retain and refer to small amounts of information that are no longer perceptually present, to guide decision-making and behaviour. Second, cognitive flexibility is the ability to switch attention and modify decision rules to adapt behaviour appropriately to changing environmental conditions. Finally, inhibitory control allows individuals to inhibit previously favoured responses that are no longer appropriate for the current conditions (Diamond 2013). Ultimately, this regulatory control is vital in allowing animals to adapt their behaviour to changing environmental conditions, a key determinant of success of individuals, populations and species under anthropogenic change (Lucon-Xiccato and Bisazza 2017a, b; Lee and Thornton 2021; Lucon-Xiccato 2022; Triki et al. 2024). The integrity of other cognitive and behavioural processes is increasingly threatened by anthropogenic environmental change (Brand et al. 2024, 2025; Martin et al. 2025a; Bertram et al. 2022), but despite mechanistic evidence that such disruptions to executive function could compromise key fitness-related behaviours (Vinogradov et al. 2025; Vaudin et al. 2022; Faria et al. 2022), this is yet to be empirically verified.

The pollution of the environment with a range of chemical contaminants has emerged as a leading threat to ecosystems worldwide (Escher et al. 2020; Tickner et al. 2020; Groh et al. 2022). Pharmaceutical pollution, in particular, is considered ubiquitous in aquatic systems (Argaluza et al. 2021; Ortúzar et al. 2022; Patel et al. 2019; Graumnitz and Jungmann 2021), and seven out of the ten most pervasive pharmaceutical pollutants are neuroactive (Wilkinson et al. 2022). These drugs target neurophysiological pathways such as the serotonergic, dopaminergic and noradrenergic systems that regulate cognition and behaviour in humans (Argaluza et al. 2021; Gould et al. 2021; López-Gil et al. 2010; Millan 2004). These pathways are evolutionarily conserved across animal taxa (Gunnarsson et al. 2008) and, as a result, neuroactive pollutants can have significant adverse effects in wildlife (Lazzara et al. 2012; Kalichak et al. 2016; de Alvarenga et al. 2017; Bókony et al. 2020; Staszny et al. 2021). Aside from direct mortality and physiological dysfunction, neuroactive pollutants can also impact cognition in non-target species (Saaristo et al. 2018; Vaudin et al. 2022; Faria et al. 2022), fundamentally altering behaviours that are critical for survival and reproductive success (Arnold et al. 2014; Aulsebrook et al. 2020; Bertram et al. 2022; Michelangeli et al. 2022). This includes predator evasion (Martin et al. 2017) and migratory movement (Brand et al. 2024, 2025), as well as social and reproductive behaviour (Brodin et al. 2013; Martin et al. 2019). Despite this, their impacts on higher-order cognitive processes remain largely unexplored (Martin et al. 2025a), and no studies to date have investigated the effects of neuroactive pharmaceutical contaminants on executive functioning specifically, in non-target wildlife. This is concerning, given that disruptions to the regulation of cognition could have serious repercussions for behavioural flexibility and its capacity to appropriately and sufficiently buffer the effects of environmental change.

In addressing this research gap, it is important that assessments of executive function accurately represent the challenges that animals face in the wild, how they cognitively resolve those challenges, and how this translates into behaviour. Conventional assessments of executive function in animals suffer from two major drawbacks. First, they were originally developed for humans and, thus, may have to be significantly adapted to be meaningful for other taxa (Kohda et al. 2019). Second, they often necessitate rule learning, extensive handling, repeated manipulation, and/or reinforcement, in a paradigm that animals would never encounter in the wild (Janmaat 2019; Boesch 2021; Salena et al. 2021). To circumvent these known issues, we employed the recently established Free Movement Pattern (FMP) Y-maze, which instead exploits an individual’s inherent motivation to explore a novel environment (Cleal et al. 2021a, b, 2023; Fontana et al. 2021). This unconditioned behavioural assay provides measures of working memory and cognitive flexibility—two of the three domains of executive functioning—without the need for repeated experimenter interventions that compromise environmental relevance.

Amitriptyline is a common antidepressant, often prescribed for mood, sleep and pain disorders (Pereira et al. 2023). It is also a widespread pollutant and is now routinely detected in surface waters globally at concentrations up to 102 ng/L (Gould et al. 2021; Wilkinson et al. 2022). Amitriptyline is a tricyclic antidepressant, modulating neural activity across the serotonergic, noradrenergic and dopaminergic systems in humans (Takahashi et al. 2012; Stott and Ang 2013; Pariyadath et al. 2016). In healthy humans, this produces broad-ranging disruptions to cognition and behaviour, including multiple aspects of executive function (van Laar et al. 2002; Iwamoto et al. 2008; Blumberg et al. 2020; Meshkat et al. 2024, Chandramouleeshwaran et al. 2024). However, it has also been found to alter neural activation across these pathways and their transcriptional profiles in aquatic species (e.g. zebrafish, *Danio rerio*; Qiu et al. 2022), with a range of neurophysiological and behavioural effects (Demin et al. 2017; Meshalkina et al. 2018; Ziarrusta et al. 2019; Liu et al. 2024). Amitriptyline can also bioaccumulate in the brain tissue of exposed fish (Ziarrusta et al. 2017), and exposure during development can downregulate genes involved in central nervous system formation (Sehonova et al. 2019). Furthermore, the pathways that amitriptyline targets are known to be important for executive function in animals as well as humans (Mueller 2012; Parker et al. 2013; Geng and Peterson 2019; Messias et al. 2016; Abreu et al. 2018; Antunes et al. 2022; Rink and Wullimann 2002, 2004). Despite this mechanistic evidence, it remains to be empirically tested how amitriptyline may affect executive function and, consequently, behaviour, at environmentally realistic concentrations. In this study, we sought to investigate whether and how exposure to environmentally relevant levels of the neuroactive pharmaceutical pollutant amitriptyline impacts executive functioning in a wild population of fish, and how this affects decision-making and ecologically relevant behaviours. Sexually mature guppies (*Poecilia reticulata*) of both sexes were collected from the wild and exposed for 10 d to one of three environmentally realistic concentrations of amitriptyline. The fish were then assessed in the FMP Y-maze to provide measures of working memory and cognitive flexibility. Based on amitriptyline’s disruptions to executive functioning in humans, the conservation of the affected neural pathways across taxa and the neurophysiological and behavioural disruptions observed in fish, we hypothesised that exposure would disrupt executive functioning in a dose-dependent manner, impacting decision-making and navigational behaviour in the FMP Y-maze.

## Materials and methods

This project followed the EthoCRED reporting recommendations for behavioural ecotoxicology studies (Bertram et al. 2025). For a systematic list of all provided criteria, see Appendix A.

### Study species

Guppies are small freshwater fish that are commonly used in ecotoxicology research. They have successfully invaded a range of ecosystems around the world due to numerous human-mediated introductions (Deacon et al. 2011; Sasanami et al. 2021) and are known to inhabit and even exploit habitats disturbed by anthropogenic activity (Widianarko et al. 2000; Araújo et al. 2009; Rolshausen et al. 2015; Gomes Silva et al. 2020). As these areas also exhibit higher environmental concentrations of pharmaceutical contaminants (Wilkinson et al. 2022), it is likely that guppies are frequently exposed to a range of pharmaceutical pollutants. Since laboratory-reared guppies have also demonstrated some executive functioning abilities in several other cognitive tasks (Macario et al. 2021; Triki et al. 2022a, b, 2023), the species is an ideal candidate to investigate the cognitive effects of pollution.

### Animal collection

Sexually mature, wild guppies of both sexes (males: *n* = 100; females: *n* = 56; total *N* = 156) were collected in October 2023 from Alligator Creek (19°26′18″ S, 146°57′01″ E), a rainforest-fed stream that runs through the Bowling Green Bay National Park in Queensland, Australia. This site has been used in previous studies as an uncontaminated source population, as water sampling indicated that it was free from contamination by other widespread pharmaceutical pollutants, known to be neuroactive (e.g. fluoxetine; Tan et al. 2020). Following collection, the fish were transported to Monash University, Victoria, Australia, and acclimated for 33 d to single-sex glass housing tanks (*n* = 39 tanks; 4 L; 15 × 15 × 25 cm; ~4 fish per tank), under a 12:12 h light: dark photoperiod (3M dotless strip light, warm white, Arlec). Single-sex housing was maintained throughout the acclimation and exposure to prevent mating from occurring. The tanks were filled with conditioned tap water (1 g/L livebearer water conditioning salt, 0.14 mL/L Nutrafin Aqua Plus water conditioner, 0.2 g/L KH generator) that was aged for a minimum of 24 h prior to use. The air temperature was monitored daily (23.5 °C ± 0.16). Each tank also contained 300 g of gravel substrate (~ 7 mm grain size), a silicone air hose, and 9 artificial leaves for enrichment. The fish were fed *ad libitum* every second day with commercial pellets (FKC Aqua Community Bites 0.8 mm Tropical Floating Pellets, Fish Keepers Choice) during acclimation and exposure. All fish were monitored daily and any clinical signs or mortality were recorded. Both the collection and experimentation were conducted in compliance with Australian law and were approved by the Monash University Animal Ethics Committee (Project ID: 37219).

### Exposure regime

Following acclimation to laboratory conditions, guppies were exposed to one of three treatments: an unexposed control (nominal concentration of 0 ng/L; *n* = 13 tanks), a low amitriptyline dose (nominal concentration of 30 ng/L; *n* = 13 tanks), or a high amitriptyline dose (nominal concentration of 300 ng/L; *n* = 13 tanks). The nominal concentrations were designed to reflect those detected in the natural environment. Specifically, amitriptyline has been detected at concentrations ranging from 1 to 102 ng/L in surface waters (Martin et al. 2025b), and up to 335 ng/L in treated wastewater that is discharged into waterways (Kasprzyk-Hordern et al. 2009). In light of this, the low nominal concentration (30 ng/L) represents a conservative but realistic level that is commonly detected in surface waters, and the high nominal concentration (300 ng/L) is more representative of heavily contaminated systems that receive direct wastewater effluents (Gould et al. 2021; Wilkinson et al. 2022). Guppies (control: *n* = 47; low amitriptyline: *n* = 50; high amitriptyline: *n* = 45) were exposed to their respective amitriptyline treatments for 10 d. This duration was based on previous studies that elicited behavioural effects in similar species (e.g. zebrafish; Ziarrusta et al. 2017; David et al. 2018; Meshalkina et al. 2018; Ziarrusta et al. 2019). A total of seven fish died during the exposure period and were therefore removed from their respective tanks (control: *n* = 3; low amitriptyline: *n* = 1; high amitriptyline: *n* = 3).

### Exposure dosing, monitoring, and analytical verification

To achieve the nominal concentrations, 612.24 µg of amitriptyline hydrochloride (98%, CAS: 549-18-8, Merck) was dissolved in 1 L of reverse-osmosis water to make a stock solution (0.6 µg/mL). This was stored in an opaque glass bottle at 2 °C to minimise degradation, alongside an identical bottle containing only reverse-osmosis water, which acted as a freshwater control. On day one of the exposure, 2 mL and 20 mL aliquots were added to each 4 L tank in the low and high amitriptyline treatments, respectively, while the control tanks received 20 mL of the freshwater control. This was followed by a secondary dose on day six, midway through the 10-day exposure, to compensate for an estimated 20% loss due to bioaccumulation in the fish and sorption to the tank itself. On day six, the tanks in the low and high amitriptyline treatments received a further 0.4 mL and 4 mL of the same stock solution, respectively, while the control tanks received 4 mL of the freshwater control. This dosing regime was designed based on previous studies showing that, in static systems, amitriptyline concentrations can fall an order of magnitude below nominal concentrations due to sorptive losses (Schmieg et al. 2022).

Water samples (50 mL) were collected from each tank on days three, six (immediately prior to the second dose), and nine of the exposure period, for analytical verification of the amitriptyline concentrations. This regime was designed to capture the dynamic range across which these concentrations may fluctuate throughout the exposure as a result of degradation, absorption by the fish and the housing apparatus, and the repeated dosage regime. A subset of the tanks were chosen to represent each treatment (control: *n*_samples_ = 9, *n*_tanks_ = 3; low: *n*_samples_ = 12, *n*_tanks_ = 4; high: *n*_samples_ = 12, *n*_tanks_ = 4 ), such that each tank had three samples, collected across the three distinct time points (total number of samples: *n* = 33). Sample selection was balanced across treatment groups, sex, position in the experimental room, and exposure days. The samples from this subset were stored at − 20 °C before being analysed individually by Envirolab Services (MPL Laboratories; NATA accreditation: 1901; accredited for compliance with ISO/IEC: 17025). Analysis was performed using liquid chromatography-tandem mass spectrometry (LC-MS/MS). There was no evidence of amitriptyline contamination in the control treatment at any point during exposure (all below the limit of quantification (LOQ); <5 ng/L, *n* = 9). The mean concentrations of amitriptyline detected in the low- and high-amitriptyline treatments were 9.25 ng/L (*SD* = 12.10, range: < LOQ–27, *n* = 12) and 309.00 ng/L (*SD* = 246.29, range: 81–760, *n* = 12), respectively. For a detailed description of the analytical protocol, see the Supplementary Material.

### Behavioural assay: the Free-Movement Pattern Y-maze

**Fig. 1:**
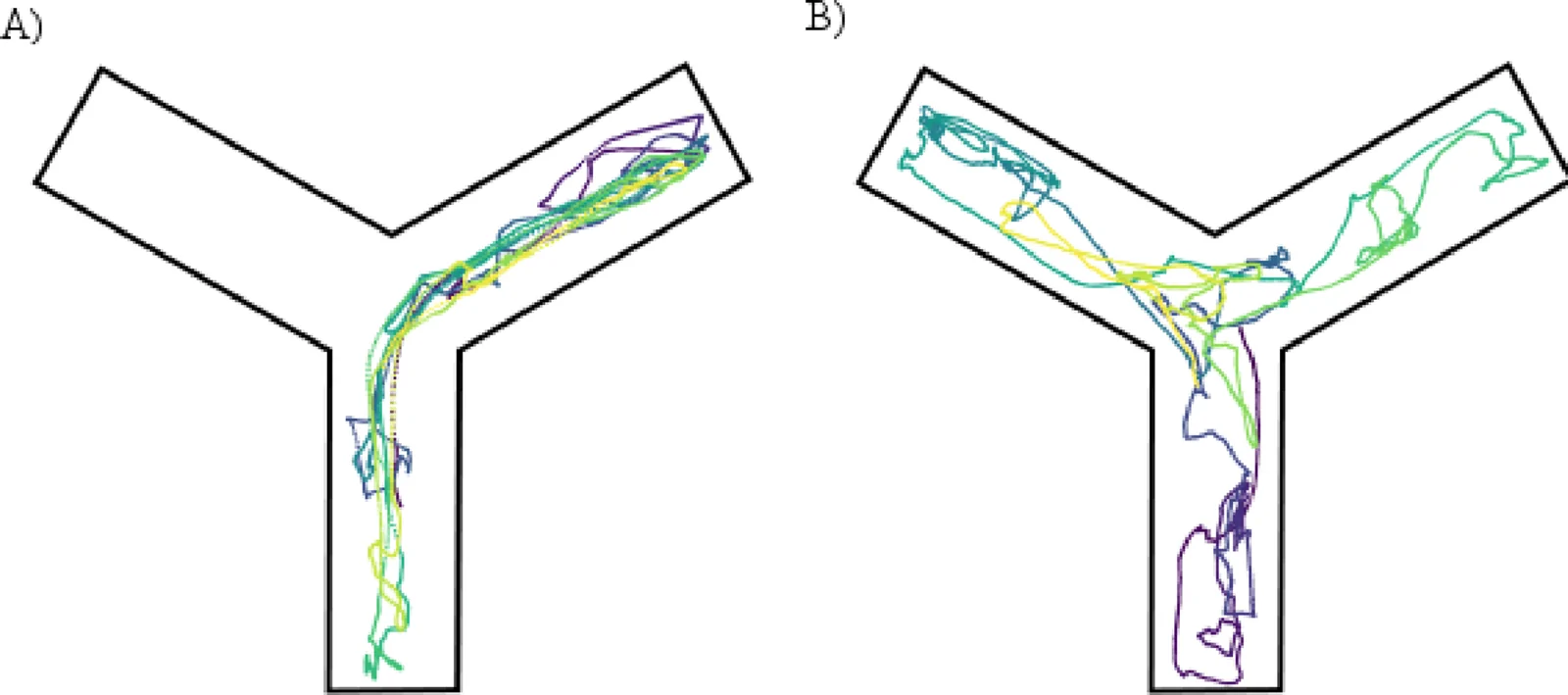
Diagram of the Free Movement Pattern Y-maze. (**A**) A fish that exhibits a characteristic preference for alternations during exploration. (**B**) A fish that exhibits a more random navigation strategy. Repeated alternations between left and right turns indicate a pattern of behaviour where the preceding three turns predict the fourth. This is considered a reliable proxy for working memory in vertebrates. Fish tracks were selected from individuals in the control treatment to exemplify each strategy and have been trimmed to a period of 10 decisions. The progression of these decisions over time is indicated by a colour gradient (yellow to purple) to distinguish the fish’s path clearly, despite retracing of the same arms

The FMP Y-maze was used to provide measures of both working memory and cognitive flexibility. In the FMP Y-maze, fish explore the novel environment of the maze, during which time they make a series of left (L) and right (R) turns between the three maze arms. This sequence is grouped into overlapping sequences of four (termed ‘tetragrams’), of which there are 16 possible combinations. This approach allows the current choice to be captured in context with the three choices that preceded it, which provides insight into the individual’s decision-making process during exploration and can reveal how past choices influence current or future behaviours (Cleal et al. 2021a). Analysis of FMP Y-maze tetragram sequences has revealed the consistent use of a particular pattern in navigational behaviour (Fig. 1). Previous research across a range of vertebrate species has shown that captive-reared animals utilise alternation sequences significantly more frequently than any of the other possible tetragrams during exploration (zebrafish, Cleal et al. 2021a, b, 2023; Fontana et al. 2021; rainbow trout, *Oncorhynchus mykiss*, Flink et al. 2025; mice, *Mus musculus*, Cleal et al. 2021b; and humans, *Homo sapiens*, Cleal et al. 2021b). Alternation sequences (i.e. LRLR and RLRL) are created when the individual switches direction at each turn, and indicate a pattern of behaviour where the preceding three turns predict the fourth. This is considered a reliable proxy for working memory (Cleal et al. 2021b). In laboratory-reared zebrafish, mice, and humans (Cleal et al. 2021b), preference for these alternation tetragrams also increases over time, as the subject becomes more familiar with the novel environment and therefore adapts their exploratory strategy. This change in strategy over time can be used as an indicator of cognitive flexibility. Furthermore, when pathways known to be important for executive functioning are pharmacologically blocked, laboratory-reared zebrafish, rats, and mice perform each tetragram indiscriminately, with equal frequency (Cleal et al. 2021a, b, 2023; Fontana et al. 2021). This apparently random decision-making endures for the entirety of the trial, further reinforcing the FMP Y-maze as a test of executive functioning (Cleal and Parker 2018; Fontana et al. 2020, 2021; Cleal et al. 2021a, b, 2023).

The FMP Y-maze has been described in detail elsewhere (Cleal et al. 2021a, b, 2023), and the protocol was adapted for this study. Forty experimental tanks were placed over an opaque white background and fitted with a Y-maze insert with 3 equidistant arms (5 × 2 × 4 cm; length × width × depth), 3D printed from white, opaque PET-G plastic. The design is available for download at: https://osf.io/jhrt5/overview?view_only=1b3c809eedde49229b23860169132b66. Fine, transparent plastic mesh (~ 2 mm hole size) was affixed to the top of the mazes with aquarium-grade silicone adhesive (Aquarium SikaSeal, Sika) to prevent the fish from jumping out during the trial. These tanks were filled to a depth of 4 cm, or just below the mesh covering. In line with previously published ecotoxicological studies (Martin et al. 2017; Bertram et al. 2022), the fish were not exposed to amitriptyline during the trial. The experimental tanks were illuminated with diffuse LED strip lighting (3M dotless strip light, warm white, Arlec), allowing the fish to orient themselves in the maze, but no explicit intra-maze cues were present.

Following 10 days of exposure, individual fish were netted from their housing tanks and placed directly into the y-maze, where they were allowed to explore the novel environment for 1 h while their behaviour was video-recorded from above (HC-V180 camcorder, Panasonic) for subsequent analysis. Each fish participated in the behavioural assay once. To accommodate the large number of fish, and because the fish were housed in groups of four, the behavioural assays were conducted across four rounds within a single day. In each round, one fish was assayed from each housing tank per round, so that by the end of the four rounds, all four fish from each tank had completed the assay. This ensured that all housing tanks were represented equally across rounds, and both round and housing tank effects could be accounted for in the statistical analyses, without round being nested within housing tank. Following the completion of each round, the fish were netted from the maze and the water was replaced to avoid cross-contamination and eliminate any conspecific cues from affecting subsequent rounds.

The movement of the fish in each video was tracked automatically using idtracker.ai, an automated AI-assisted software for quantitative analysis of animal behaviour (Romero-Ferrero et al. 2019). To ensure consistency between tracks and across treatments, the area of interest (the perimeter of the maze walls) and the tracking settings for each video were specified by an observer who was blinded to the amitriptyline treatment. Pixel coordinates representing the position of the fish in every video frame were recorded and assigned to predetermined polygonal zones of the maze to determine the occupied arm. The tracked trajectory of each individual was plotted to verify tracking accuracy, and tracking parameters were adjusted where necessary. Transitions between zones were converted into a time series of left and right turns. The turns were grouped into tetragrams, and assigned one of the 16 possible types, based on the turn sequence (e.g. LLLL, LLLR, et cetera). The positional coordinates of each fish were used to calculate the total distance travelled during the trial (cm), as an independent measure of general activity.

### Morphological measurements

Immediately following the completion of the behavioural trials, the fish were netted from the experimental tanks and humanely killed in MS-222 solution (~ 250 mg/L). All individuals were photographed (Powershot S120 digital camera, Canon) at 90° to the lateral plane with standardised lighting and camera conditions. From the photographs, their standard length (from the tip of the snout to the caudal peduncle) was measured using Fiji software (v. 2.14.0) (Schindelin et al. 2012) for inclusion in the statistical analyses.

### Statistical analyses

All data analysis was performed in R (version 4.4.1) using RStudio (R Core Team, 2024). Bayesian generalised linear mixed-effects models were fit for all response variables using the *brms* package (Bürkner 2017). All models were run across four chains for 4000 iterations (1000 warmup iterations) using weakly informative priors (normal distributions with µ = 0 and SD = 10 were specified for fixed effects and all other priors were left at the default settings in the brms which constrain parameters to only possible values, e.g. positive standard deviation). The final models were re-run with non-informative priors to confirm that inferences were robust to prior specification. Posterior predictive checks were performed to ensure adequate model fit, and sufficient model convergence was confirmed through inspection of the Gelman-Rubin diagnostic statistic (all $\:\widehat{R}\:$= 1.00). The *emmeans* package (Lenth 2025) was used to summarise posterior estimates as estimated marginal means and to compute pairwise contrasts between tetragram types and treatment groups. These contrasts and the associated 95% credible intervals (CrIs) were used to determine the magnitude and direction of any differences between groups. CrIs that excluded zero were interpreted as evidence of meaningful differences.

To determine the frequency with which guppies performed each of the 16 possible tetragrams during exploration, we modelled the number of times a tetragram was performed with a zero-inflated negative binomial distribution (logit link). Fixed effects included tetragram type (e.g. LLLL, LLLR, etc), treatment (control, low-amitriptyline, or high-amitriptyline), total number of turns a fish made during the trial (centred and scaled), sex (male or female), trial round (as a factor with levels 1–4) and standard length of the fish (centred and scaled), as well as the two-way interactions between tetragram type and treatment, and tetragram type and total turns. Unique individual fish ID, nested within exposure tank, was included as a random effect, to account for individual variation and the group exposure design. Expected Log Predictive Density (ELPD) estimated via leave-one-out cross-validation (LOO-CV) confirmed that the inclusion of tetragram type as a predictor substantially improved model predictive performance (ΔELPD = − 275.32 ± 29.96), suggesting that a meaningful amount of variation in tetragram count can be explained by differences among tetragram types. In the resultant model, if a tetragram’s type meaningfully predicts the number of times it was performed, this would indicate a preference for certain tetragrams over others, which necessitates the involvement of working memory.

Having identified that alternations (i.e. LRLR and RLRL) were the predominant tetragrams used by guppies (see *Results*), we modelled the proportion of alternations (relative to the total number of tetragrams each fish performed) with a Beta distribution (logit link). A small positive coefficient (0.00001) was added to these proportions to accommodate a single individual that performed no alternations. Fixed effects included treatment, total turns completed (centred and scaled), sex, standard length (centred and scaled), and the interaction between treatment and total turns (centred and scaled). Exposure tank was included as a random effect. In this context, a greater proportion of alternations (relative to all other tetragrams) would indicate a greater reliance on this repeating pattern of behaviour during exploration. This provides a measure of working memory. Posterior draws from this model were used to compare the involvement of working memory in exploration between different treatment groups.

To determine whether the aforementioned reliance on alternations was consistent throughout the trial, or whether this pattern emerged or changed over time, we divided the 1-h trial into six 10-min time bins. We modelled counts of each tetragram within each bin with a zero-inflated Poisson distribution (logit link). Fixed effects included tetragram type, treatment, total number of turns a fish made during the trial (centred and scaled), sex, trial round, and standard length of the fish (centred and scaled), as well as the interaction between tetragram type, time bin, and treatment. Unique individual fish ID, nested within exposure tank, was included as a random effect. Estimated marginal trends were derived from the posterior distributions using the *emmeans* package (Lenth 2025). Here, any temporal trend in the frequency at which guppies performed alternations would indicate the adjustment of behaviour with experience, which requires a degree of cognitive flexibility. We compared the slope of these temporal trends to evaluate differences in cognitive flexibility between treatment groups.

## Results

### Working memory

**Fig. 2:**
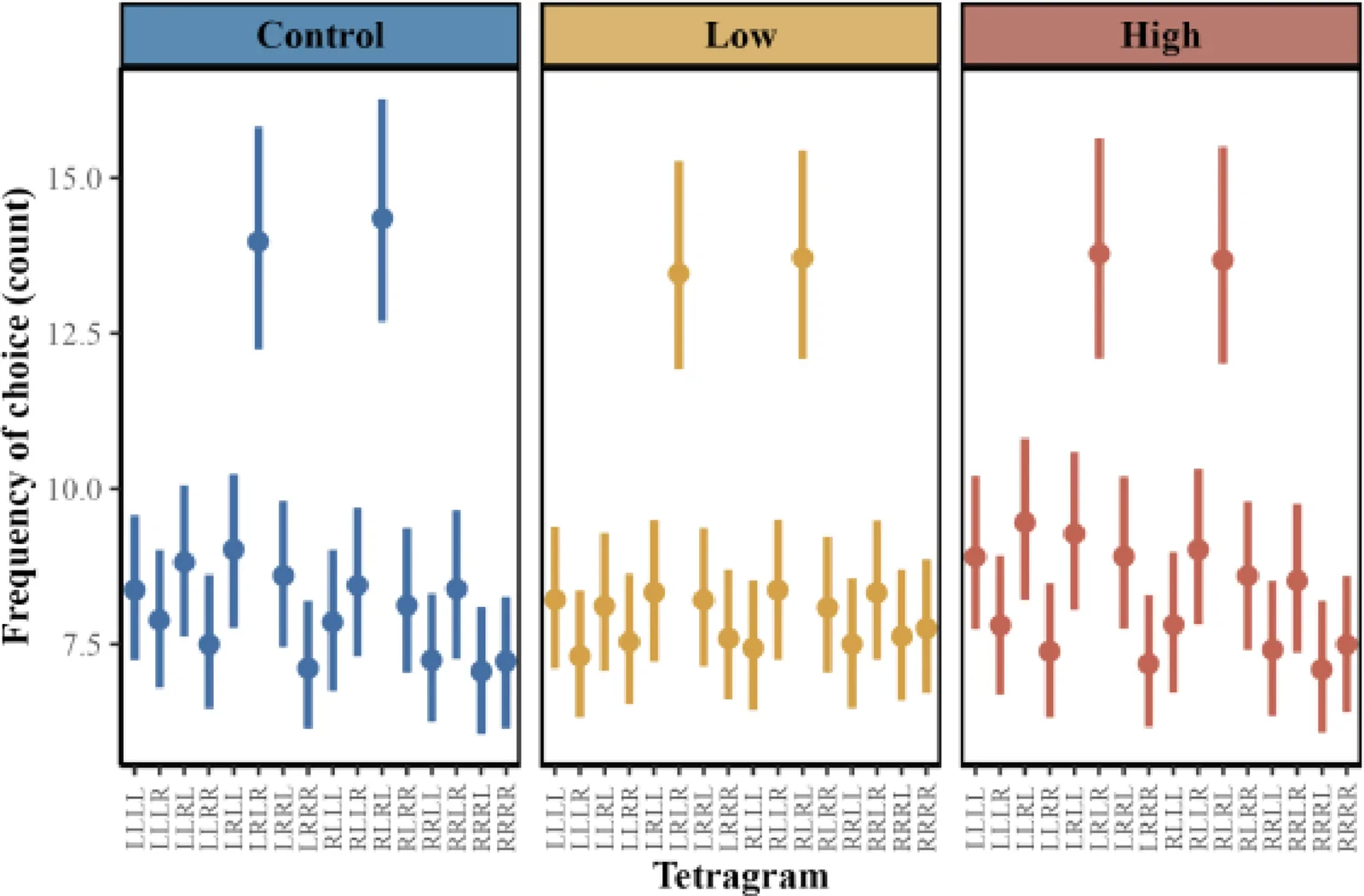
Frequency of global tetragram usage during 1-h of FMP Y-maze exploration, for fish exposed to one of three amitriptyline treatments: control (0 ng/L, *n* = 47; blue), low (9 ng/L, *n* = 50; yellow), or high (309 ng/L, *n* = 45; red). Points represent the model-estimated marginal means of the number of times a fish performed each of the 16 possible tetragrams, and error bars represent the 95% credible intervals

**Fig. 3:**
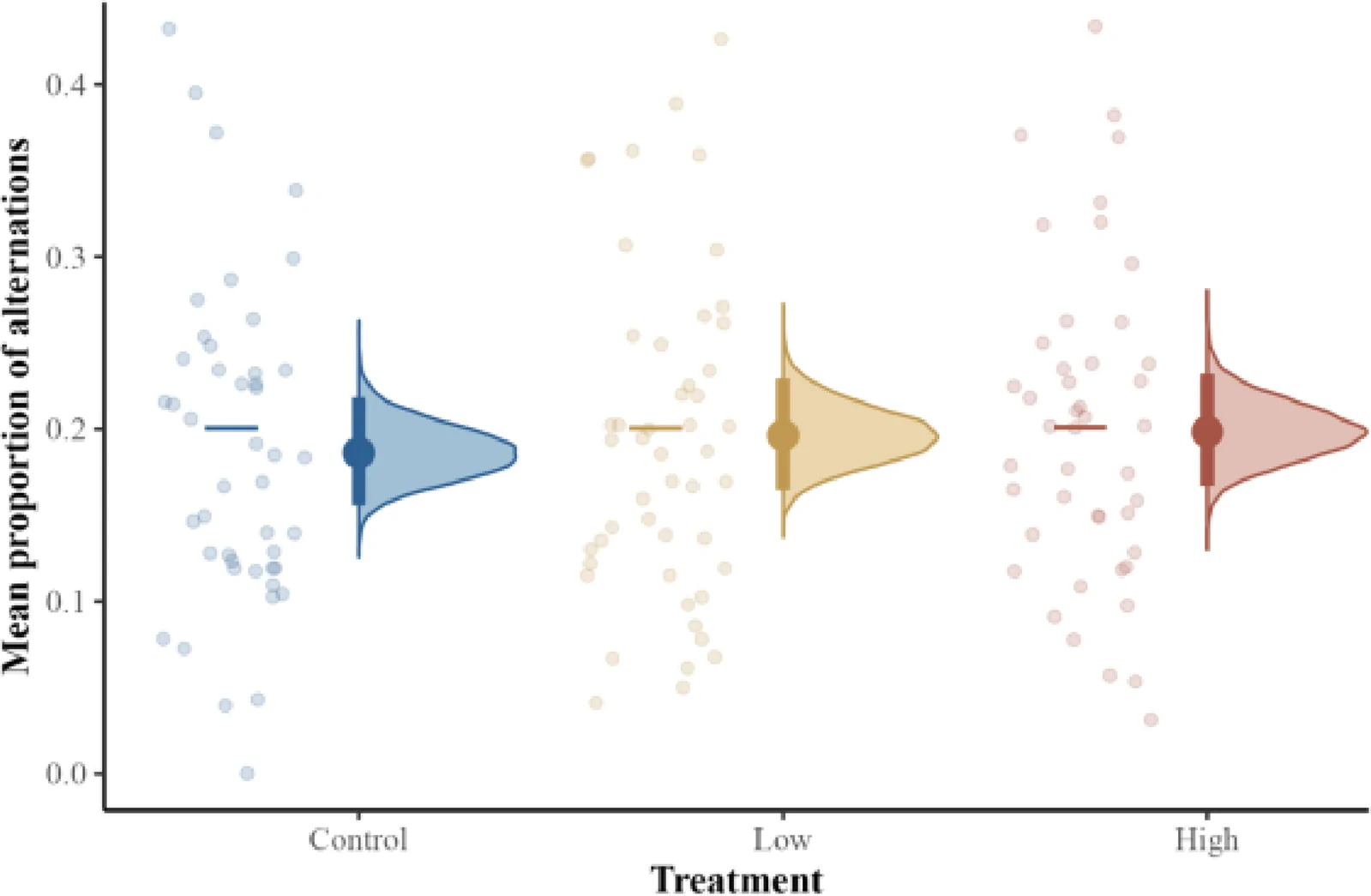
Alternation (i.e. LRLR and RLRL) usage during FMP Y-maze exploration. Results are given as a proportion of the total number of tetragrams completed during 1-h of exploration, for fish exposed to three amitriptyline treatments: control (0 ng/L, *n* = 47; blue), low (9 ng/L, *n* = 50; yellow), or high (309 ng/L, *n* = 45; red). The left side of each treatment displays the raw data in a scatterplot, with horizontal lines representing the raw means. The right side displays model estimates. The points represent the model-estimated marginal means, error bars represent the 95% credible intervals and density plots represent the posterior distribution

In all treatment groups, fish exhibited an exploratory strategy that was overrepresented by non-random sequences of alternating left and right turns (i.e. LRLR and RLRL; “alternations”). On average, control fish performed the two alternation tetragrams 14.19 times each during a trial [95% CrI: 12.81, 15.55], while the 14 other tetragrams occurred 7.99 times each [CrI: 7.46, 8.49] (Fig. 2; Table S5). Control fish performed 6.20 more alternations (CrI: 5.02, 7.46) than other tetragrams, a difference of 55.75% (CrI: 47.91, 63.79) (Figure S2; Table S6). Fish exhibited this involvement of working memory in directing exploration across all treatment groups (Fig. 3; Tables S5 and S6). In all treatments, fish used alternations at a meaningfully higher frequency than other tetragrams (Fig. 3; Table S8). Alternation tetragrams made up 18.6% (15.9, 21.8) of all the tetragrams used by control fish. Low-amitriptyline fish used alternations 19.6% (16.5, 22.9) of the time, and high-amitriptyline fish used them 19.8% (16.7, 23.2) of the time (Fig. 3; Table S8). However, there were no meaningful differences between treatments in the proportion of alternations used, relative to other tetragrams. In other words, all treatment groups exhibited the same preference for alternations, but amitriptyline exposure did not have a meaningful effect on the strength of this preference. Therefore, there was no evidence that amitriptyline exposure disrupted working memory (Fig. 3; Table S9).

### Cognitive flexibility

**Fig. 4:**
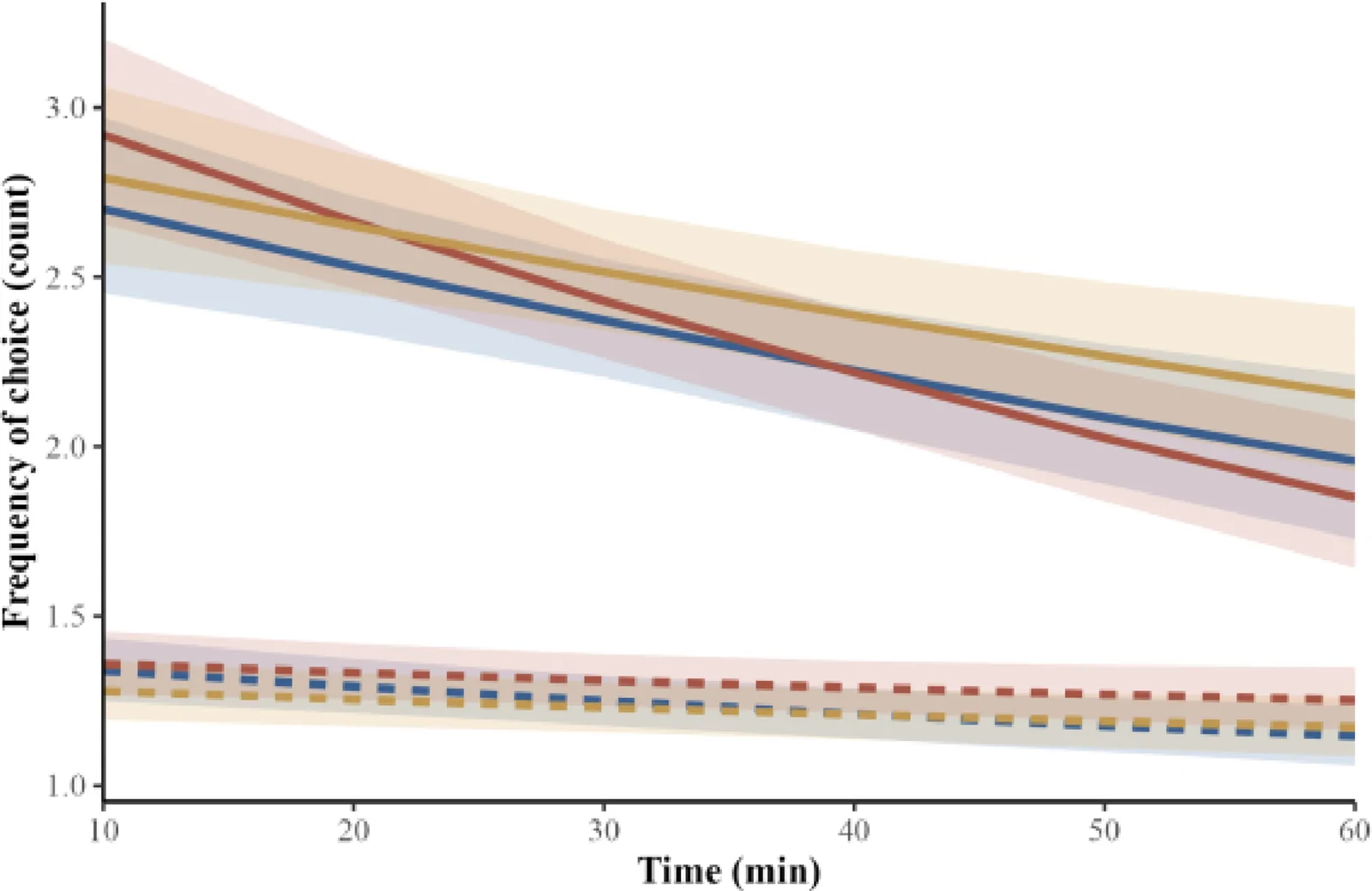
Adjustment of exploratory strategy in fish exposed to three amitriptyline treatments: control (0 ng/L, *n* = 47; blue), low amitriptyline (9 ng/L, *n* = 50; yellow), or high amitriptyline (309 ng/L, *n* = 45; red), by strategy, over 1-h of FMP Y-maze exploration. The mean number of alternation tetragrams that fish performed (i.e. LRLR and RLRL; solid line) and the mean number of the remaining 14 other tetragrams (dashed line) are displayed for each 10-min time bin of the trial, for each treatment group. The shaded regions represent 95% credible intervals

Across all treatment groups, guppies displayed a clear involvement of cognitive flexibility in exploration, as they adjusted their exploratory behaviour with experience. The number of times that control guppies performed both alternations, and the other tetragrams, decreased with each passing 10-minute time bin (Fig. 4; Tables S11 and S12). The average number of alternations declined from 2.65 [2.38, 2.89] in the first 10 min of the 1-h trial to 1.92 [1.68, 2.15] in the final 10 min (Table S11). For the other tetragrams, occurrence also declined, from 1.31 [1.23, 1.41] other tetragrams in the first 10 min to 1.12 [1.04, 1.21] in the final 10 min (Table S12). When contrasted against one another, the rate at which these adjustments occurred was markedly different between alternations and the other tetragrams, with alternations declining at a meaningfully faster rate (Fig. 4). Specifically, guppies in the control treatment performed, on average, 0.145 [CrI: 0.075, 0.215] fewer alternations with every bin, and 0.037 [CrI: 0.016, 0.057] fewer other tetragrams (Table S13). This equates to a difference of 0.107 less alternations than other tetragrams [CrI: 0.036, 0.180] with every additional 10 min exploring the maze (Table S14). This indicates that the decline in alternations is meaningfully different to any general decrease in activity throughout the trial (Figure S3), which would affect all other tetragrams indiscriminately, and instead attributable to cognitive flexibility allowing behavioural adjustment to the novel environment. However, there was no evidence that amitriptyline exposure affected cognitive flexibility (Fig. 4). Both the low- and high-exposed fish also exhibited a similar decline in tetragram use over time (Table S13), with a steeper decline in alternations compared to other tetragrams (Table S14). Crucially, these rates of decline—both for alternations and other tetragrams—did not differ between treatments (Table S15). Thus, although alternation use decreased more rapidly than other tetragrams within each treatment, this difference was consistent across treatment groups. In other words, the meaningful distinction between the general reduction in activity observed in the other tetragrams, and the reduction of alternation tetragrams specifically, remained robust to amitriptyline exposure.

## Discussion

Here, we investigated whether and how executive function manifests in decision-making and behaviour in a wild population of guppies, and whether this was disrupted by exposure to environmentally realistic concentrations of the widespread neuroactive pollutant amitriptyline. We found evidence that two of the three domains of executive function actively regulate cognition during exploration of a novel environment and that this can be clearly detected in repeating patterns of navigation behaviour that adapt with experience. We also found that neither working memory nor cognitive flexibility were affected by short-term exposure to two environmentally realistic concentrations of amitriptyline.

These results present the first evidence that executive function can regulate behaviour in wild fish. During exploration, the control guppies relied predominantly on a pattern of behaviour in which they alternate repeatedly between successive left and right turns. This illustrates that future turns are (in part) predicted by those that precede them, which indicates the involvement of working memory to direct decision-making during exploration. Previous studies have identified the preference for alternations during Y-maze exploration as a reliable measure of working memory in captive-reared fish (Fontana et al. 2020, 2021; Cleal et al. 2021a, 2023; Flink et al. 2025), as well as in other vertebrates such as mice and humans (Cleal et al. 2021b). However, this is the first demonstration of working memory in a wild fish population. Furthermore, the only evidence of working memory in guppies comes from a highly controlled object permanence task. Here, working memory was observed exclusively in lines artificially selected for increased relative telencephalon size over five generations, and wild-type guppies were unable to demonstrate working memory in this context (Triki et al. 2023). In contrast, our findings show that working memory is an intrinsic component of decision-making in fish that directly supports navigation and exploratory behaviours that are highly relevant for survival in the wild.

The predominance of alternations in exploratory strategy also changed over the course of the trial, as fish adjusted their behaviour with increasing experience. This is a clear indicator of cognitive flexibility (Cleal et al. 2021a, b, 2023; Fontana et al. 2021), which allows animals to adapt their behaviour to changes in the environment (Tomasek et al. 2023, 2024; Triki et al. 2024). The FMP Y-maze represents a novel, initially unpredictable environment. As an individual explores, they learn more information about the maze that can inform behaviour. This allows them to adjust their navigational strategy accordingly, both to the initial novelty and subsequently as they become more familiar and the environment becomes more predictable (Cleal et al. 2021b). Early in exploration, control fish exhibited a very high predominance of alternations initially, relative to other tetragram types. However, this declined sharply with time, such that by the end of the trial alternations were used as often as other tetragrams. Although the occurrence of other tetragram types also decreased over the course of the trial, these changes were less pronounced, as their initial frequencies were already relatively low. Consequently, the overall reduction in activity observed across the trial was driven primarily by the pronounced decline in alternations rather than a uniform decrease across all tetragrams. This distinction indicates that the reduction in alternation use cannot be explained solely by a general decline in activity, but instead reflects the involvement of cognitive flexibility in adjusting exploration, as individuals accumulate information about their environment.

Previous studies show that laboratory-reared zebrafish, mice, and humans all display some degree of cognitive flexibility in the FMP Y-maze by increasing their preference for alternations over time (Cleal et al. 2021a, b, 2023). This contrasts with the decrease in frequency that we observed in guppies. There are several possible explanations for this difference. The first is that this is simply species-dependent. This is unlikely because guppies are much more similar, both phylogenetically and cognitively, to zebrafish than zebrafish are to humans, and yet they seem to share fewer similarities in how their behaviour develops over time. Another possible explanation for these differences is that there are often cognitive differences between wild and lab-reared animals (Näslund and Johnsson 2016). In the wild, the ability to appropriately adapt behaviour to constantly updating information about the environment is a key determinant of survival and reproductive success (Sih et al. 2011; Sih 2013; Snell-Rood and Steck 2019; Namboodiri et al. 2016; Reader 2015; Jackson et al. 2020). Animals face a range of cognitive challenges in the wild, such as searching for food or mates in patchy resource distributions and maintaining complex anti-predator behaviour like shoaling. These challenges are absent in a lab environment and therefore selection on cognitive ability can be significantly relaxed (Reznick and Ghalambor 2005; Salena et al. 2021). In addition to the loss of this evolutionary driver, plastic cognitive changes can occur in response to simplified environments within an individual’s lifetime (Silva et al. 2023). Reduced environmental complexity limits brain growth (Kihslinger and Nevitt 2006; Näslund et al. 2012), neuronal proliferation (Dunlap et al. 2011), as well as synaptogenesis and neural plasticity (Cardona et al. 2022). This can occur over periods as short as a week in zebrafish (von Krogh et al. 2010), with corresponding impacts on cognitive abilities like foraging in a novel environment, predator avoidance, and spatial navigation (Salvanes et al. 2013). As these results are the first indicator of how a wild population of animals uses cognitive flexibility to develop exploratory strategies in the FMP Y-maze, it is unsurprising that they differ cognitively and behaviourally to laboratory-reared zebrafish. Our reliance on model species and the relative dearth of studies assessing cognition in wild animals (Salena et al. 2021; Bshary and Triki 2022; Vila-Pouca et al. 2025) make it difficult to determine if the previously ‘universal’ strategy observed in other species is actually an artefact of relaxed selection on cognition in captivity. Our results suggest that cognitive differences between captive-reared and wild-caught animals may affect how behaviour like exploration is expressed, which is highly relevant for individual fitness.

Housing or experimental environments that lack sufficient stimuli can present a significant confound in behavioural studies, affecting attention and cognition, and redirecting exploration and goal-directed behaviour (Meier et al. 2024; Mieske et al. 2022). The maze environment is devoid of any enrichment, as the lack of intra-maze cues is necessary to avoid biasing exploratory behaviour. However, predictability, confinement and monotony in experimental conditions is associated with boredom in other animals, including fur-farmed mink (*Mustela vison*; Meagher and Mason 2012, Meagher et al. 2017), laboratory rats (*Rattus norvegicus domestica*), mice (Mieske et al. 2022), and ferrets (*Mustela furo*; Burn et al. 2020), as well as several fish species (Atlantic salmon, *Salmo salar*; Salvanes et al. 2013; gilthead bream, *Sparus aurata*; Oliveira et al. 2024; and zebrafish; Birgersson et al. 2024). Boredom arises when both internal and external stimulation are insufficient to maintain optimum arousal, and this can produce a range of altered behavioural states (Mieske et al. 2022, Meagher 2018, Burn 2017). These range from increases in time spent awake but inactive, to stimulus-seeking or stereotypic behaviour as animals lack sufficient sensory and cognitive stimuli. Natural environments offer increased environmental complexity compared to laboratory conditions, which improve cognitive abilities in fish. This includes spatial (Salvanes et al. 2013, Abreu et al. 2019) and social learning (Strand et al. 2010), as well as behavioural flexibility demonstrated in visual discrimination (Brunet et al. 2023) and spatial tasks (Hesse et al. 2019; Ahlbeck Bergendahl et al. 2016; Salvanes et al. 2013), The latter in particular is associated with increases in cell proliferation and upregulation at the telencephalon specifically, the neural substrate for executive functioning abilities (Abreu et al. 2019; Salvanes et al. 2013). On the other hand, fish raised in laboratory environments with reduced enrichment exhibit inflexible and limited cognitive and behavioural repertoires in comparison (Cardona et al. 2022, Salvanes et al. 2013; Abreu et al. 2019). It is possible that the wild guppies used in this study possess greater cognitive flexibility than the laboratory-raised zebrafish used in previous studies, which may explain the differences we observed. Our study was modelled on the 1-h duration used in zebrafish, where an increase in alternation use was observed at 10 min intervals up to the 40-min mark, when it then begins to decline for the final 20 min (Cleal et al. 2021b, 2023). Conversely, wild guppies exhibit a strong preference for alternations initially, which declines over time. This seems to indicate that, at some point, the maze starts to become predictable or familiar, and fish stop actively exploring, which manifests as a decline in alternation use and activity in general (von Krogh et al. 2010; Birgersson et al. 2024). One disadvantage of an interval of 10 min is that if guppies adapt their strategy more rapidly than zebrafish, these changes are undetectable (Cleal et al. 2021b). These gaps highlight the potential pitfalls in designing species-specific variations in the duration or temporal resolution of a cognitive task, based on preconceived notions of how cognitively advanced each species is, especially when the goal is to make comparisons among species.

In this study, we found no effect of short-term amitriptyline exposure on either working memory or cognitive flexibility in adult guppies. Previous studies have found that amitriptyline has a range of effects on neurophysiology and behaviour in several fish species. Environmentally realistic concentrations produce oxidative stress, alterations to energy metabolism and brain monoamine concentrations in adult zebrafish (Demin et al. 2017; Meshalkina et al. 2018; Tang et al. 2025; Qiu et al. 2022) and gilthead bream (Ziarrusta et al. 2017, 2019). If zebrafish are exposed to such concentrations as embryos, amitriptyline can affect gene expression related to brain, eye, heart, and bone development, which is associated with perturbation to swimming behaviour in adults (Liu et al. 2024). Developmental exposure to environmentally realistic levels of amitriptyline can also cause morphological abnormalities, affect gene expression, enzyme metabolism and nervous system formation, and cause cardiotoxicity in larval zebrafish (Sehonova et al. 2019), three-spined sticklebacks (*Gasterosteus aculeatus*), brown trout (*Salmo trutta*), and common carp (*Cyprinus carpio*) (Gould et al. 2021, 2024). In zebrafish, the aforementioned neurochemical changes are accompanied by effects on locomotion, feeding behaviour, aggression, shoaling and anxiety-like behaviour into adulthood (Gould et al. 2021, 2024). Fish exposed to amitriptyline as adults exhibit similar neurochemical disturbances to brain monoamine levels, however, behavioural effects on locomotion, shoaling, feeding, and anxiety behaviour only become apparent at concentrations exceeding environmental levels or longer-term exposure periods up to or exceeding 3 w in duration (Gould et al. 2021, 2024). While this is consistent with our findings, it is important to note that pharmaceutical pollution is a growing concern. The concentrations of these contaminants in the environment are only rising, and these drugs are effectively pseudo-persistent in the environment due to their continuous release (Wilkinson et al. 2022). Therefore, wild animals are exposed to pharmaceutical contaminants constantly, throughout all developmental stages. It remains unclear whether guppies are genuinely resilient to the effects of amitriptyline on executive functioning, or whether this may conceal underlying shifts in brain monoamine chemistry that could influence cognition and behaviour, particularly when exposed long-term or at critical neurodevelopmental windows, as would occur in the wild.

In conclusion, these findings illustrate that executive function is instrumental in shaping behaviour that is highly ecologically relevant in the wild, and clearly distinguishable in FMP Y-maze performance. Demonstrating cognitive flexibility and working memory in a simple, ecologically relevant navigation task that is easily implemented and highly translatable across species opens a valuable opportunity to investigate executive functioning in wild populations, which remain comparatively understudied. While we did not detect any effects of acute exposure to environmentally realistic concentrations of amitriptyline on executive functioning and navigational behaviour, it is important to interpret this result with the context that in the wild, animals are exposed to neuroactive pollutants constantly, throughout all stages of development. Given the importance of executive functioning, and cognition more generally, in allowing animals to appropriately produce and maintain such behaviours that are critical for survival, it is imperative that we better understand how neuroactive pharmaceutical pollutants like amitriptyline affect cognition, and how this may manifest in behaviour.

## Supplementary Information

Below is the link to the electronic supplementary material.

Supplementary Material 1

Supplementary Material 2

## Data Availability

Data and R code associated with the statistical analyses performed are available on OSF at the following URL: https:/osf.io/jhrt5/overview? view_only=1b3c809eedde49229b23860169132b66.

## References

[CR1] Abreu MS, Messias JPM, Thörnqvist P-O, Winberg S, Soares MC (2018) Monoaminergic levels at the forebrain and diencephalon signal for the occurrence of mutualistic and conspecific engagement in client reef fish. Sci Rep 8(1):7346. 10.1038/s41598-018-25513-6 29743658 PMC5943261

[CR2] Abreu CC, Fernandes TN, Henrique EP, Pereira PDC, Marques SB, Herdeiro SLS, Oliveira FRR, Magalhães NGM, Anthony DC, Melo MAD, Guerreiro-Diniz C, Diniz DG, Picanço-Diniz CW (2019) Small-scale environmental enrichment and exercise enhance learning and spatial memory of *Carassius auratus*, and increase cell proliferation in the telencephalon: an exploratory study. Braz J Med Biol Res 52(5):e8026. 10.1590/1414-431X20198026 31038577 PMC6487742

[CR3] Ahlbeck Bergendahl I, Salvanes AGV, Braithwaite VA (2016) Determining the effects of duration and recency of exposure to environmental enrichment. Appl Anim Behav Sci 176:163–169. 10.1016/j.applanim.2015.11.002

[CR4] Antunes DF, Soares MC, Taborsky M (2022) Dopamine modulates social behaviour in cooperatively breeding fish. Mol Cell Endocrinol 550:111649. 10.1016/j.mce.2022.111649 35436519

[CR5] Araújo F, Peixoto M, Pinto B, Teixeira T (2009) Distribution of guppies *Poecilia reticulata* (Peters, 1860) and *Phalloceros caudimaculatus* (Hensel, 1868) along a polluted stretch of the Paraíba do Sul River, Brazil. Braz J Biol 69(1):41–48. 10.1590/s1519-69842009000100005 19347144

[CR6] Argaluza J, Domingo-Echaburu S, Orive G, Medrano J, Hernandez R, Lertxundi U (2021) Environmental pollution with psychiatric drugs. World J Psychiatry 11(10):791–804. 10.5498/wjp.v11.i10.791 34733642 PMC8546762

[CR7] Arnold KE, Brown AR, Ankley GT, Sumpter JP (2014) Medicating the environment: assessing risks of pharmaceuticals to wildlife and ecosystems. Philos Trans R Soc Lond B Biol Sci 369(1656):20130569. 10.1098/rstb.2013.0569 25405959 PMC4213582

[CR8] Aulsebrook LC, Bertram MG, Martin JM, Aulsebrook AE, Brodin T, Evans JP, Hall MD, O’Bryan MK, Pask AJ, Tyler CR, Wong BBM (2020) Reproduction in a polluted world: implications for wildlife. Reproduction 160(2):13–23. 10.1530/REP-20-0154 32442963

[CR11] Bertram MG, Martin JM, McCallum ES, Alton LA, Brand JA, Brooks BW, Cerveny D, Fick J, Ford AT, Hellström G, Michelangeli M, Nakagawa S, Polverino G, Saaristo M, Sih A, Tan H, Tyler CR, Wong BBM, Brodin T (2022) Frontiers in quantifying wildlife behavioural responses to chemical pollution. Biol Rev 97(4):1346–1364. 10.1111/brv.12844 35233915 PMC9543409

[CR10] Bertram MG, Ågerstrand M, Thoré ESJ, Allen J, Balshine S, Brand JA, Brooks BW, Dang Z, Duquesne S, Ford AT, Hoffmann F, Hollert H, Jacob S, Kloas W, Klüver N, Lazorchak J, Ledesma M, Maack G, Macartney EL, Martin JM, Melvin SD, Michelangeli M, Mohr S, Padilla S, Pyle G, Saaristo M, Sahm R, Smit E, Steevens JA, van den Berg S, Vossen LE, Wlodkowic D, Wong BBM, Ziegler M, Brodin T (2025) EthoCRED: a framework to guide reporting and evaluation of the relevance and reliability of behavioural ecotoxicity studies. Biol Rev 100(2):556–585. 10.1111/brv.13154 39394884 PMC11885694

[CR9] Bertram MG, Ågerstrand M, Balshine S, Brand JA, Brooks BW, Dang Z, Ford AT, Hollert H, LeFauve MK, Manera JL, Martin JM, Michelangeli M, Moiron M, Moore ER, Puglis HJ, Sih A, Steevens JA, Thoré ESJ, Wong BBM, Zink L, Brodin T (2026) Are behavioral ecotoxicity endpoints relevant at the population level? Evidence-based insights for environmental protection. Enviro Sci Technol 60(1):86–95. 10.1021/acs.est.5c07777 PMC12810249 41437732

[CR12] Birgersson L, Odenlund S, Sturve J (2024) Effects of environmental enrichment on exposure to human-relevant mixtures of endocrine disrupting chemicals in zebrafish. Anim (Basel) 14(9):1296. 10.3390/ani14091296 PMC11083384 38731300

[CR13] Blumberg MJ, Vaccarino SR, McInerney SJ (2020) Procognitive effects of antidepressants and other therapeutic agents in major depressive disorder: a systematic review. J Clin Psychiatry 81(4):19r13200. 10.4088/JCP.19r13200 32726521

[CR14] Boesch C (2021) Identifying animal complex cognition requires natural complexity. iScience 24(3):102195. 10.1016/j.isci.2021.102195 33733062 PMC7937571

[CR15] Bókony V, Verebélyi V, Ujhegyi N, Mikó Z, Nemesházi E, Szederkényi M, Orf S, Vitányi E, Móricz ÁM (2020) Effects of two little-studied environmental pollutants on early development in anurans. Environ Pollut 260:114078. 10.1016/j.envpol.2020.114078 32041031

[CR16] Brand JA, Bertram MG, Cerveny D, McCallum ES, Hellström G, Michelangeli M, Palm D, Brodin T (2024) Psychoactive pollutant alters movement dynamics of fish in a natural lake system. Proc Biol Sci. 291(2036): 20241760. 10.1098/rspb.2024.1760 PMC11631415 39657799

[CR17] Brand JA, Michelangeli M, Shry SJ, Moore ER, Bose APH, Cerveny D, Martin JM, Hellström G, McCallum ES, Holmgren A, Thoré ESJ, Fick J, Brodin T, Bertram MG (2025) Pharmaceutical pollution influences river-to-sea migration in Atlantic salmon (*Salmo salar*). Science 388(6743):217–222. 10.1126/science.adp7174 40208989

[CR18] Brodin T, Fick J, Jonsson M, Klaminder J (2013) Dilute concentrations of a psychiatric drug alter behavior of fish from natural populations. Science 339(6121):814–815. 10.1126/science.1226850 23413353

[CR19] Brunet V, Lafond T, Kleiber A, Lansade L, Calandreau L, Colson V (2023) Environmental enrichment improves cognitive flexibility in rainbow trout in a visual discrimination task: first insights. Front Vet Sci 10:1184296. 10.3389/fvets.2023.1184296 37396987 PMC10313407

[CR20] Bshary R, Triki Z (2022) Fish ecology and cognition: insights from studies on wild and wild-caught teleost fishes. Curr Opin Behav Sci 46:101174. 10.1016/j.cobeha.2022.101174

[CR21] Bürkner P-C (2017) brms: An R package for Bayesian multilevel models using Stan. J Stat Softw 80(1):1–28. 10.18637/jss.v080.i01

[CR22] Burn CC (2017) Bestial boredom: a biological perspective on animal boredom and suggestions for its scientific investigation. Anim Behav 130:141–151. 10.1016/j.anbehav.2017.06.006

[CR23] Burn CC, Raffle J, Bizley JK (2020) Does ‘playtime’ reduce stimulus-seeking and other boredom-like behaviour in laboratory ferrets? Anim Welf 29(1):19–26. 10.7120/09627286.29.1.019 32226239 PMC7099939

[CR131] Cardona E, Brunet V, Baranek E, Milhade L, Skiba-Cassy S, Bobe J, Calandreau L, Roy J, Colson V (2022) Physical enrichment triggers brain plasticity and influences blood plasma circulating miRNA in rainbow trout (*Oncorhynchus mykiss*). Biology (Basel) 11(8):1093 10.3390/biology11081093 35892949 PMC9394377

[CR24] Chandramouleeshwaran S, Khan WU, Inglis F, Rajji TK (2024) Impact of psychotropic medications on cognition among older adults: a systematic review. Int Psychogeriatr 36(12):1110–1127. 10.1017/S1041610223000844 37860872

[CR28] Cleal M, Parker MO (2018) Moderate developmental alcohol exposure reduces repetitive alternation in a zebrafish model of fetal alcohol spectrum disorders. Neurotoxicol Teratol 70:1–9. 10.1016/j.ntt.2018.09.001 30201482

[CR25] Cleal M, Fontana BD, Double M, Mezabrovschi R, Parcell L, Redhead E, Parker MO (2021a) Dopaminergic modulation of working memory and cognitive flexibility in a zebrafish model of aging-related cognitive decline. Neurobiol Aging 102:1–16. 10.1016/j.neurobiolaging.2021.02.005 33676049

[CR27] Cleal M, Fontana BD, Ranson DC, McBride SD, Swinny JD, Redhead E, Parker MO (2021b) The Free-movement pattern Y-maze: A cross-species measure of working memory and executive function. Behav Res Methods 53(2):536–557. 10.3758/s13428-020-01452-x 32748238 PMC8062322

[CR26] Cleal M, Fontana BD, Hillman C, Parker MO (2023) Ontogeny of working memory and behavioural flexibility in the free movement pattern (FMP) Y-maze in zebrafish. Behav Processes. 212:104943. 10.1016/j.beproc.2023.104943 37689254

[CR29] David A, Lange A, Tyler CR, Hill EM (2018) Concentrating mixtures of neuroactive pharmaceuticals and altered neurotransmitter levels in the brain of fish exposed to a wastewater effluent. Sci Total Environ 621:782–790. 10.1016/j.scitotenv.2017.11.265 29202289

[CR30] de Alvarenga KAF, Sacramento EK, Rosa DV, Souza BR, de Rezende VB, Romano-Silva MA (2017) Effects of antipsychotics on intestinal motility in zebrafish larvae. Neurogastroenterol Motil 29:e13006. 10.1111/nmo.13006 27981679

[CR31] Deacon AE, Ramnarine IW, Magurran AE (2011) How reproductive ecology contributes to the spread of a globally invasive fish. PLoS ONE 6(9):e24416. 10.1371/journal.pone.0024416 21957449 PMC3176282

[CR32] Demin KA, Kolesnikova TO, Khatsko SL, Meshalkina DA, Efimova EV, Morzherin YY, Kalueff AV (2017) Acute effects of amitriptyline on adult zebrafish: potential relevance to antidepressant drug screening and modeling human toxidromes. Neurotoxicol Teratol 62:27–33. 10.1016/j.ntt.2017.04.002 28438663

[CR33] Diamond A (2013) Executive functions. Annu Rev Psychol 64:135–168. 10.1146/annurev-psych-113011-143750 23020641 PMC4084861

[CR34] Dunlap KD, Silva AC, Chung M (2011) Environmental complexity, seasonality and brain cell proliferation in a weakly electric fish, *Brachyhypopomus gauderio*. J Exp Biol 214(5):794–805. 10.1242/jeb.051037 21307066 PMC3036548

[CR35] Escher BI, Stapleton HM, Schymanski EL (2020) Tracking complex mixtures of chemicals in our changing environment. Science 367:388–392. 10.1126/science.aay6636 31974244 PMC7153918

[CR36] Faria M, Bellot M, Soto O, Prats E, Montemurro N, Manjarrés D, Gómez-Canela C, Raldúa D (2022) Developmental exposure to sertraline impaired zebrafish behavioral and neurochemical profiles. Front Physiol 13:1040598. 10.3389/fphys.2022.1040598 36467683 PMC9716079

[CR37] Flink H, Berge A, Leggieri F, Kolm N, Tibblin P (2025) Transient cognitive impacts of oxygen deprivation caused by catch-and-release angling. Biol Lett 21:20240527. 10.1098/rsbl.2024.0527 39809327 PMC11732411

[CR39] Fontana BD, Cleal M, Parker MO (2020) Female adult zebrafish (*Danio rerio*) show higher levels of anxiety-like behavior than males, but do not differ in learning and memory capacity. Eur J Neurosci 52:2604–2613. 10.1111/ejn.14588 31597204

[CR38] Fontana BD, Cleal M, Gibbon AJ, McBride SD, Parker MO (2021) The effects of two stressors on working memory and cognitive flexibility in zebrafish (*Danio rerio*): The protective role of D1/D5 agonist on stress responses. Neuropharmacology 196:108681. 10.1016/j.neuropharm.2021.108681 34175323

[CR40] Geng Y, Peterson RT (2019) The zebrafish subcortical social brain as a model for studying social behavior disorders. Dis Model Mech 12(8):039446. 10.1242/dmm.039446 PMC6737945 31413047

[CR41] Gomes Silva G, Eric C, Wronski T, Riesch R, Apio A, Plath M (2020) Water pollution affects fish community structure and alters evolutionary trajectories of invasive guppies (*Poecilia reticulata*). Sci Total Environ 730:138912. 10.1016/j.scitotenv.2020.138912 32402962

[CR42] Gould SL, Winter MJ, Norton WHJ, Tyler CR (2021) The potential for adverse effects in fish exposed to antidepressants in the aquatic environment. Environ Sci Technol 55:16299–16312. 10.1021/acs.est.1c04724 34856105

[CR43] Gould SL, Winter MJ, Trznadel M, Lange A, Hamilton CM, Boreham RJ, Hetheridge MJ, Young A, Norton WHJ, Tyler CR (2024) Exposure effects of environmentally relevant concentrations of the tricyclic antidepressant amitriptyline in early life stage zebrafish. Environ Sci Technol 58:13194–13204. 10.1021/acs.est.3c08126 39018108 PMC11295126

[CR44] Graumnitz S, Jungmann D (2021) The database Pharmaceuticals in the Environment. Report No. FB000627/ENG, German Environment Agency

[CR45] Groh K, vom Berg C, Schirmer K, Tlili A (2022) Anthropogenic chemicals as underestimated drivers of biodiversity loss: scientific and societal implications. Environ Sci Technol 56(2):707–710. 10.1021/acs.est.1c08399 34974706

[CR46] Gunnarsson L, Jauhiainen A, Kristiansson E, Nerman O, Larsson DGJ (2008) Evolutionary conservation of human drug targets in organisms used for environmental risk assessments. Environ Sci Technol 42(15):5807–5813. 10.1021/es8005173 18754513

[CR47] Hesse S, Sandmann S, Bakker TCM, Thünken T (2019) Impact of social rearing-environment on performance in a complex maze in females of a cichlid fish. Behav Processes. 167:103915. 10.1016/j.beproc.2019.103915 31349022

[CR48] Iwamoto K, Takahashi M, Nakamura Y, Kawamura Y, Ishihara R, Uchiyama Y, Ebe K, Noda A, Noda Y, Yoshida K, Iidaka T, Ozaki N (2008) The effects of acute treatment with paroxetine, amitriptyline, and placebo on driving performance and cognitive function in healthy Japanese subjects: a double-blind crossover trial. Hum Psychopharmacol 23(5):399–407. 10.1002/hup.939 18383000

[CR49] Jackson BJ, Fatima GL, Oh S, Gire DH (2020) Many paths to the same goal: balancing exploration and exploitation during probabilistic route planning. eNeuro 7(3):0536–0519. 10.1523/ENEURO.0536-19.2020 PMC7294469 32414790

[CR50] Janmaat KRL (2019) What animals do not do or fail to find: a novel observational approach for studying cognition in the wild. Evol Anthropol 28:303–320. 10.1002/evan.21794 31418959 PMC6916178

[CR51] Kalichak F, Idalencio R, Rosa JGS, de Oliveira TA, Koakoski G, Gusso D, de Abreu MS, Giacomini ACV, Barcellos HHA, Fagundes M, Piato AL, Barcellos LJG (2016) Waterborne psychoactive drugs impair the initial development of zebrafish. Environ Toxicol Pharmacol 41:89–94. 10.1016/j.etap.2015.11.014 26667671

[CR52] Kasprzyk-Hordern B, Dinsdale RM, Guwy AJ (2009) The removal of pharmaceuticals, personal care products, endocrine disruptors and illicit drugs during wastewater treatment and its impact on the quality of receiving waters. Water Res 43:363–380. 10.1016/j.watres.2008.10.047 19022470

[CR53] Kihslinger RL, Nevitt GA (2006) Early rearing environment impacts cerebellar growth in juvenile salmon. J Exp Biol 209(3):504–509. 10.1242/jeb.02019 16424100

[CR54] Kohda M, Hotta T, Takeyama T, Awata S, Tanaka H, Asai J-y, Jordan AL (2019) If a fish can pass the mark test, what are the implications for consciousness and self-awareness testing in animals? PLoS Biol 17(2):e3000021. 10.1371/journal.pbio.3000021 30730878 PMC6366756

[CR55] Lazzara R, Blázquez M, Porte C, Barata C (2012) Low environmental levels of fluoxetine induce spawning and changes in endogenous estradiol levels in the zebra mussel Dreissena polymorpha. Aquat Toxicol 106–107:123–130. 10.1016/j.aquatox.2011.11.003 22155424

[CR56] Lee VE, Thornton A (2021) Animal cognition in an urbanised world. Front Ecol Evol 9:633947. 10.3389/fevo.2021.633947 34409044 PMC7611524

[CR57] Lenth RV, Piaskowski J (2025) emmeans: estimated marginal means, aka least-squares means. See https://rvlenth.github.io/emmeans/

[CR58] Liu A, Chen C, Chen K, Shi Y, Grabowski RC, Qiu X (2024) Effects of parental exposure to amitriptyline on the survival, development, behavior, and gene expression in zebrafish offspring. Sci Total Environ 912:169173. 10.1016/j.scitotenv.2023.169173 38064809

[CR59] Logue SF, Gould TJ (2014) The neural and genetic basis of executive function: attention, cognitive flexibility, and response inhibition. Pharmacol Biochem Behav 123:45–54. 10.1016/j.pbb.2013.08.007 23978501 PMC3933483

[CR60] López-Gil X, Artigas F, Adell A (2010) Unraveling monoamine receptors involved in the action of typical and atypical antipsychotics on glutamatergic and serotonergic transmission in prefrontal cortex. Curr Pharm Des 16(5):502–515. 10.2174/138161210790361416 19909228

[CR61] Lucon-Xiccato T (2022) The contribution of executive functions to sex differences in animal cognition. Neurosci Biobehav Rev 138:104705. 10.1016/j.neubiorev.2022.104705 35605792

[CR62] Lucon-Xiccato T, Bisazza A (2017a) Individual differences in cognition among teleost fishes. Behav Processes. 141:184–195. 10.1016/j.beproc.2017.01.015 28126389

[CR63] Lucon-Xiccato T, Bisazza A (2017b) Sex differences in spatial abilities and cognitive flexibility in the guppy. Anim Behav 123:53–60. 10.1016/j.anbehav.2016.10.026

[CR67] Martin JM, Saaristo M, Bertram MG, Lewis PJ, Coggan TL, Clarke BO, Wong BBM (2017) The psychoactive pollutant fluoxetine compromises antipredator behaviour in fish. Environ Pollut 222:592–599. 10.1016/j.envpol.2016.10.010 28063712

[CR64] Macario A, Darden SK, Verbruggen F, Croft DP (2021) Intraspecific variation in inhibitory motor control in guppies, *Poecilia reticulata*. J Fish Biol 98:317–328. 10.1111/jfb.14608 33128393

[CR128] Martin JM, Bertram MG, Saaristo M, Ecker TE, Hannington SL, Tanner JL, Michelangeli M, O'Bryan MK, Wong BBM (2019) Impact of the widespread pharmaceutical pollutant fluoxetine on behaviour and sperm traits in a freshwater fish. Sci Total Environ 650:1771-1778 10.1016/j.scitotenv.2018.09.294 30278421

[CR65] Martin JM, Michelangeli M, Bertram MG, Blanchfield PJ, Brand JA, Brodin T, Brooks BW, Cerveny D, Fergusson KN, Lagisz M, Lovin LM, Ligocki IY, Nakagawa S, Ozeki S, Sandoval-Herrera N, Scarlett KR, Sundin J, Tan H, Thoré ESJ, Wong BBM, McCallum ES (2025a) Evidence of the impacts of pharmaceuticals on aquatic animal behaviour (EIPAAB): a systematic map and open access database. Environ Evid. 14:4. 10.1186/s13750-025-00357-6 PMC11924672 40108717

[CR66] Martin JM, Brand JA, McCallum ES (2025b) Aligning behavioural ecotoxicology with real-world water concentrations: current minimum tested levels for pharmaceuticals far exceed environmental reality. Environ Sci Technol Lett 12:1308–1313. 10.1021/acs.estlett.5c00665

[CR125] Meagher RK, Mason GJ (2012) Environmental enrichment reduces signs of boredom in caged mink. PLoS one 7(11):e49180 10.1371/journal.pone.0049180 23155462 PMC3498363

[CR126] Meagher RK, Campbell DLM, Mason GJ (2017) Boredom-like states in mink and their behavioural correlates: a replicate study. Appl Anim Behav Sci 197:112-119 10.1016/j.applanim.2017.08.001

[CR127] Meagher RK (2018) Is boredom an animal welfare concern? Anim Welf 28(1):21-32 10.7120/09627286.28.1.021

[CR68] Meier M, Martarelli CS, Wolff W (2024) Is boredom a source of noise and/or a confound in behavioral science research? Humanit Soc Sci Commun 11:368. 10.1057/s41599-024-02851-7

[CR69] Meshalkina DA, Kysil EV, Antonova KA, Demin KA, Kolesnikova TO, Khatsko SL, Gainetdinov RR, Alekseeva PA, Kalueff AV (2018) The effects of chronic amitriptyline on zebrafish behavior and monoamine neurochemistry. Neurochem Res 43:1191–1199. 10.1007/s11064-018-2536-5 29740748

[CR70] Messias JP, Santos TP, Pinto M, Soares MC (2016) Stimulation of dopamine D₁ receptor improves learning capacity in cooperating cleaner fish. Proc R Soc B 283:20152272. 10.1098/rspb.2015.2272 26791613 PMC4795016

[CR71] Michelangeli M, Martin JM, Pinter-Wollman N, Ioannou CC, McCallum ES, Bertram MG, Brodin T (2022) Predicting the impacts of chemical pollutants on animal groups. Trends Ecol Evol 37:789–802. 10.1016/j.tree.2022.05.009 35718586

[CR132] Mieske P, Hobbiesiefken U, Fischer-Tenhagen C, Heinl C, Hohlbaum K, Kahnau P, Meier J, Wilzopolski J, Butzke D, Rudeck J, Lewejohann L, Diederich K (2022) Bored at home?—A systematic review on the effect of environmental enrichment on the welfare of laboratory rats and mice. Front Vet Sci 9:899219. 10.3389/fvets.2022.899219 PMC9435384 36061113

[CR72] Millan MJ (2004) The role of monoamines in the actions of established and novel antidepressant agents: a critical review. Eur J Pharmacol 500(1):371–384. 10.1016/j.ejphar.2004.07.038 15464046

[CR73] Mueller T (2012) What is the thalamus in zebrafish? Front Neurosci 6:64. 10.3389/fnins.2012.00064 22586363 PMC3345571

[CR74] Namboodiri VMK, Levy JM, Mihalas S, Sims DW, Hussain Shuler MG (2016) Rationalizing spatial exploration patterns of wild animals and humans through a temporal discounting framework. Proc Natl Acad Sci USA 113:8747–8752. 10.1073/pnas.1601664113 27385831 PMC4978240

[CR76] Näslund J, Johnsson JI (2016) Environmental enrichment for fish in captive environments: effects of physical structures and substrates. Fish Fish 17:1–30. 10.1111/faf.12088

[CR75] Näslund J, Aarestrup K, Thomassen ST, Johnsson JI (2012) Early enrichment effects on brain development in hatchery-reared Atlantic salmon (*Salmo salar*): no evidence for a critical period. Can J Fish Aquat Sci 69:1481–1490. 10.1139/f2012-074

[CR77] Oliveira AR, Cabrera-Álvarez MJ, Soares F, Diáz-Gil C, Candeias-Mendes A, Saraiva JL, Arechavala-Lopez P (2024) Structural enrichment promotes natural behaviour and welfare of captive gilthead seabream broodstock. Appl Anim Behav Sci 275:106289. 10.1016/j.applanim.2024.106289

[CR78] Ortúzar M, Esterhuizen M, Olicón-Hernández DR, González-López J, Aranda E (2022) Pharmaceutical pollution in aquatic environments: a concise review of environmental impacts and bioremediation systems. Front Microbiol 13:869332. 10.3389/fmicb.2022.869332 35558129 PMC9087044

[CR79] Pariyadath V, Gowin JL, Stein EA (2016) Chapter 8 - Resting state functional connectivity analysis for addiction medicine: From individual loci to complex networks. In: Ekhtiari H, Paulus MP (eds) Progress in Brain Research. Elsevier, pp 155–173. 10.1016/bs.pbr.2015.07.015 26822358

[CR80] Parker M, Brock A, Walton R, Brennan C (2013) The role of zebrafish (*Danio rerio*) in dissecting the genetics and neural circuits of executive function. Front Neural Circuits 7:63. 10.3389/fncir.2013.00063 23580329 PMC3619107

[CR81] Patel M, Kumar R, Kishor K, Mlsna T, Pittman CU Jr, Mohan D (2019) Pharmaceuticals of emerging concern in aquatic systems: chemistry, occurrence, effects, and removal methods. Chem Rev 119:3510–3673. 10.1021/acs.chemrev.8b00299 30830758

[CR82] Pereira CDS, Cruz JN, Ferreira MKM, Baia-da-Silva DC, Fontes-Junior EA, Lima RR (2023) Global research trends and hotspots analysis of the scientific production of amitriptyline: a bibliometric approach. Pharmaceuticals (Basel) 16:1047. 10.3390/ph16071047 37513958 PMC10386017

[CR83] Qiu X, Chen C, Shi Y, Chen K, Li M, Xu H, Wu X, Takai Y, Shimasaki Y, Oshima Y (2022) Persistent impact of amitriptyline on the behavior, brain neurotransmitter, and transcriptional profile of zebrafish (*Danio rerio*). Aquat Toxicol 245:106129. 10.1016/j.aquatox.2022.106129 35248893

[CR130] R Core Team (2024) R: A language and environment for statistical computing. R Foundation for Statistical Computing, Vienna, Austria. https://www.R-project.org/

[CR84] Reader SM (2015) Causes of individual differences in animal exploration and search. Top Cogn Sci 7:451–468. 10.1111/tops.12148 25982255

[CR85] Reznick DN, Ghalambor CK (2005) Selection in nature: experimental manipulations of natural populations. Integr Comp Biol 45:456–462. 10.1093/icb/45.3.456 21676790

[CR86] Rink E, Wullimann MF (2002) Development of the catecholaminergic system in the early zebrafish brain: an immunohistochemical study. Dev Brain Res 137:89–100. 10.1016/s0165-3806(02)00354-1 12128258

[CR87] Rink E, Wullimann MF (2004) Connections of the ventral telencephalon (subpallium) in the zebrafish (*Danio rerio*). Brain Res 1011:206–220. 10.1016/j.brainres.2004.03.027 15157807

[CR88] Rolshausen G, Phillip DA, Beckles DM, Akbari A, Ghoshal S, Hamilton PB, Tyler CR, Scarlett AG, Ramnarine I, Bentzen P, Hendry AP (2015) Do stressful conditions make adaptation difficult? Guppies in the oil-polluted environments of southern Trinidad. Evol Appl 8(9):854–870. 10.1111/eva.12289 26495039 PMC4610383

[CR89] Romero-Ferrero F, Bergomi M, Hinz R, Heras F, Polavieja G (2019) idtracker.ai: tracking all individuals in large collectives of unmarked animals. Nat Methods 16(2):179–182. 10.1038/s41592-018-0295-5 30643215

[CR90] Saaristo M, Brodin T, Balshine S, Bertram MG, Brooks BW, Ehlman SM, McCallum ES, Sih A, Sundin J, Wong BBM, Arnold KE (2018) Direct and indirect effects of chemical contaminants on the behaviour, ecology and evolution of wildlife. Proc R Soc B 285(1885):20181297. 10.1098/rspb.2018.1297 30135169 PMC6125903

[CR91] Salena MG, Turko AJ, Singh A, Pathak A, Hughes E, Brown C, Balshine S (2021) Understanding fish cognition: a review and appraisal of current practices. Anim Cogn 24(3):395–406. 10.1007/s10071-021-01488-2 33595750

[CR92] Salvanes AG, Moberg O, Ebbesson LO, Nilsen TO, Jensen KH, Braithwaite VA (2013) Environmental enrichment promotes neural plasticity and cognitive ability in fish. Proc R Soc B 280(1767):20131331. 10.1098/rspb.2013.1331 23902903 PMC3735255

[CR93] Sasanami M, Hustedt J, Alexander N, Horstick O, Bowman L, Hii J, Echaubard P, Braack L, Overgaard HJ (2021) Does anthropogenic introduction of guppy fish (*Poecilia reticulata*) impact faunal species diversity and abundance in natural aquatic habitats? A systematic review protocol. Environ Evid 10:33. 10.1186/s13750-021-00248-6

[CR94] Schindelin J, Arganda-Carreras I, Frise E, Kaynig V, Longair M, Pietzsch T, Preibisch S, Rueden C, Saalfeld S, Schmid B, Tinevez J-Y, White DJ, Hartenstein V, Eliceiri K, Tomancak P, Cardona A (2012) Fiji: an open-source platform for biological-image analysis. Nat Methods 9(7):676–682. 10.1038/nmeth.2019 22743772 PMC3855844

[CR129] Schmieg H, Krais S, Kübler K, Ruhl AS, Schmidgall IM, Zwiener C, Köhler HR, Triebskorn R (2022) Effects of the antidepressant amitriptyline on juvenile brown trout and their modulation by microplastics. Toxics 10(12): 763 10.3390/toxics10120763 36548596 PMC9787892

[CR95] Sehonova P, Hodkovicova N, Urbanova M, Örn S, Blahova J, Svobodova Z, Faldyna M, Chloupek P, Briedikova K, Carlsson G (2019) Effects of antidepressants with different modes of action on early life stages of fish and amphibians. Environ Pollut 254:112999. 10.1016/j.envpol.2019.112999 31404734

[CR98] Sih A (2013) Understanding variation in behavioural responses to human-induced rapid environmental change: a conceptual overview. Anim Behav 85(5):1077–1088. 10.1016/j.anbehav.2013.02.017

[CR97] Sih A (2024) Behavioural ecology for a changing world. In: Wong BBM, Candolin U (eds) Behavioural Responses to a Changing World: Challenges and Applications, 1st edn. Oxford University Press, USA, pp 329–345

[CR99] Sih A, Ferrari MC, Harris DJ (2011) Evolution and behavioural responses to human-induced rapid environmental change. Evol Appl 4(2):367–387. 10.1111/j.1752-4571.2010.00166.x 25567979 PMC3352552

[CR100] Silva PF, Garcia de Leaniz C, Freire FAM, Silveira VAM, Luchiari AC (2023) Different housing conditions for zebrafish: what are the effects? Behav Processes. 209:104886. 10.1016/j.beproc.2023.104886 37150333

[CR101] Snell-Rood EC, Steck MK (2019) Behaviour shapes environmental variation and selection on learning and plasticity: review of mechanisms and implications. Anim Behav 147:147–156. 10.1016/j.anbehav.2018.08.007

[CR102] Staszny A, Dobosy P, Maasz G, Szalai Z, Jakab G, Pirger Z, Szeberenyi J, Molnar E, Pap LO, Juhasz V, Weiperth A, Urbanyi B, Kondor AC, Ferincz A (2021) Effects of pharmaceutically active compounds (PhACs) on fish body and scale shape in natural waters. PeerJ 9:e10642. 10.7717/peerj.10642 33614266 PMC7882141

[CR103] Stott SRW, Ang SL (2013) The generation of midbrain dopaminergic neurons. In: Rubenstein JLR, Rakic P (eds) Patterning and Cell Type Specification in the Developing CNS and PNS, 2nd edn. Academic, Oxford, pp 435–453

[CR104] Strand DA, Utne-Palm AC, Jakobsen PJ, Braithwaite VA, Jensen KH, Salvanes AGV (2010) Enrichment promotes learning in fish. Mar Ecol Prog Ser 412:273–282. 10.3354/meps08682

[CR105] Takahashi H, Yamada M, Suhara T (2012) Functional significance of central D1 receptors in cognition: beyond working memory. J Cereb Blood Flow Metab 32(7):1248–1258. 10.1038/jcbfm.2011.194 22234338 PMC3390810

[CR106] Tan H, Polverino G, Martin JM, Bertram MG, Wiles SC, Palacios MM, Bywater CL, White CR, Wong BBM (2020) Chronic exposure to a pervasive pharmaceutical pollutant erodes among-individual phenotypic variation in a fish. Environ Pollut 263:114450. 10.1016/j.envpol.2020.114450 32283454

[CR107] Tang J, Liu A, Chen K, Shi Y, Qiu X (2025) Exposure to amitriptyline disturbs behaviors in adult zebrafish and their offspring via altering neurotransmitter levels. Comp Biochem Physiol C Toxicol Pharmacol 288:110079. 10.1016/j.cbpc.2024.110079 39551226

[CR108] Tickner D, Opperman JJ, Abell R, Acreman M, Arthington AH, Bunn SE, Cooke SJ, Dalton J, Darwall W, Edwards G, Harrison I, Hughes K, Jones T, Leclère D, Lynch AJ, Leonard P, McClain ME, Muruven D, Olden JD, Ormerod SJ, Robinson J, Tharme RE, Thieme M, Tockner K, Wright M, Young L (2020) Bending the curve of global freshwater biodiversity loss: an emergency recovery plan. Bioscience 70(4):330–342. 10.1093/biosci/biaa002 32284631 PMC7138689

[CR110] Tomasek M, Stark M, Dufour V, Jordan A (2023) Cognitive flexibility in a Tanganyikan bower-building cichlid, *Aulonocranus dewindti*. Anim Cogn 26:1959–1971. 10.1007/s10071-023-01830-w 37851187 PMC10770232

[CR109] Tomasek M, Soller K, Dufour V, Jordan A (2024) Differences in inhibitory control in two species of Tanganyikan bower-building cichlids contrasting in building flexibility. Ecol Evol 14:e11406. 10.1002/ece3.11406 38846708 PMC11154817

[CR113] Triki Z, Fong S, Amcoff M, Kolm N (2022a) Artificial mosaic brain evolution of relative telencephalon size improves inhibitory control abilities in the guppy (*Poecilia reticulata*). Evolution 76(1):128–138. 10.1111/evo.14405 34806770

[CR114] Triki Z, Granell-Ruiz M, Fong S, Amcoff M, Kolm N (2022b) Brain morphology correlates of learning and cognitive flexibility in a fish species (*Poecilia reticulata*). Proc R Soc B. 289(1978):20220844. 10.1098/rspb.2022.0844 PMC9277233 35858069

[CR112] Triki Z, Fong S, Amcoff M, Vàsquez-Nilsson S, Kolm N (2023) Experimental expansion of relative telencephalon size improves the main executive function abilities in guppy. PNAS Nexus 2(6):129. 10.1093/pnasnexus/pgad129 PMC10281379 37346268

[CR111] Triki Z, Zhou T, Argyriou E, Sousa de Novais E, Servant O, Kolm N (2024) Social complexity affects cognitive abilities but not brain structure in a Poecilid fish. Behav Ecol 35(3):arae026. 10.1093/beheco/arae026 38638166 PMC11025466

[CR115] van Laar MW, Volkerts ER, Verbaten MN, Trooster S, van Megen HJ, Kenemans JL (2002) Differential effects of amitriptyline, nefazodone and paroxetine on performance and brain indices of visual selective attention and working memory. Psychopharmacology 162(4):351–363. 10.1007/s00213-002-1116-0 12172688

[CR116] Vaudin P, Augé C, Just N, Mhaouty-Kodja S, Mortaud S, Pillon D (2022) When pharmaceutical drugs become environmental pollutants: potential neural effects and underlying mechanisms. Environ Res 205:112495. 10.1016/j.envres.2021.112495 34883077

[CR117] Vila-Pouca C, De Waele H, Parsékian A, Erroi S, De Rooij M, Labohm E, Deacon A, Kotrschal A (2025) A novel apparatus for studying fish cognition in the wild. Methods Ecol Evol 16(5):939–948. 10.1111/2041-210X.70002

[CR118] Vinogradov IM, Fox RJ, Fichtel C, Kappeler PM, Jennions MD (2025) Paternity analysis reveals sexual selection on cognitive performance in mosquitofish. Nat Ecol Evol 9:692–704. 10.1038/s41559-025-02645-3 40000808

[CR119] von Krogh K, Sørensen C, Nilsson GE, Øverli Ø (2010) Forebrain cell proliferation, behavior, and physiology of zebrafish, *Danio rerio*, kept in enriched or barren environments. Physiol Behav 101:32–39. 10.1016/j.physbeh.2010.04.003 20385156

[CR120] Widianarko B, Van Gestel CAM, Verweij RA, Van Straalen NM (2000) Associations between trace metals in sediment, water, and guppy, *Poecilia reticulata* (Peters), from urban streams of Semarang, Indonesia. Ecotoxicol Environ Saf 46(1):101–107. 10.1006/eesa.1999.1879 10806000

[CR121] Wilkinson JL, Boxall ABA, Kolpin DW, Leung KMY, Lai RWS, Galbán-Malagón C, Adell AD, Mondon J, Metian M, Marchant RA, Bouzas-Monroy A, Cuni-Sanchez A, Coors A, Carriquiriborde P, Rojo M, Gordon C, Cara M, Moermond M, Luarte T, Petrosyan V, Perikhanyan Y, Mahon CS, McGurk CJ, Hofmann T, Kormoker T, Iniguez V, Guzman-Otazo J, Tavares JL, Gildasio De Figueiredo F, Razzolini MTP, Dougnon V, Gbaguidi G, Traoré O, Blais JM, Kimpe LE, Wong M, Wong D, Ntchantcho R, Pizarro J, Ying G-G, Chen C-E, Páez M, Martínez-Lara J, Otamonga J-P, Poté J, Ifo SA, Wilson P, Echeverría-Sáenz S, Udikovic-Kolic N, Milakovic M, Fatta-Kassinos D, Ioannou-Ttofa L, Belušová V, Vymazal J, Cárdenas-Bustamante M, Kassa BA, Garric J, Chaumot A, Gibba P, Kunchulia I, Seidensticker S, Lyberatos G, Halldórsson HP, Melling M, Shashidhar T, Lamba M, Nastiti A, Supriatin A, Pourang N, Abedini A, Abdullah O, Gharbia SS, Pilla F, Chefetz B, Topaz T, Yao KM, Aubakirova B, Beisenova R, Olaka L, Mulu JK, Chatanga P, Ntuli V, Blama NT, Sherif S, Aris AZ, Looi LJ, Niang M, Traore ST, Oldenkamp R, Ogunbanwo O, Ashfaq M, Iqbal M, Abdeen Z, O’Dea A, Morales-Saldaña JM, Custodio M, de la Cruz H, Navarrete I, Carvalho F, Gogra AB, Koroma BM, Cerkvenik-Flajs V, Gombač M, Thwala M, Choi K, Kang H, Ladu JLC, Rico A, Amerasinghe P, Sobek A, Horlitz G, Zenker AK, King AC, Jiang J-J, Kariuki R, Tumbo M, Tezel U, Onay TT, Lejju JB, Vystavna Y, Vergeles Y, Heinzen H, Pérez-Parada A, Sims DB, Figy M, Good D, Teta C (2022) Proc Natl Acad Sci USA 119(8):e2113947119 Pharmaceutical pollution of the world’s rivers. 10.1073/pnas.2113947119 35165193 PMC8872717

[CR122] Wong BBM, Candolin U (2014) Behavioral responses to changing environments. Behav Ecol 26(3):665–673. 10.1093/beheco/aru183

[CR123] Ziarrusta H, Mijangos L, Izagirre U, Plassmann MM, Benskin JP, Anakabe E, Olivares M, Zuloaga O (2017) Bioconcentration and biotransformation of amitriptyline in gilt-head bream. Environ Sci Technol 51(4):2464–2471. 10.1021/acs.est.6b05831 28106990

[CR124] Ziarrusta H, Ribbenstedt A, Mijangos L, Picart-Armada S, Perera-Lluna A, Prieto A, Izagirre U, Benskin JP, Olivares M, Zuloaga O, Etxebarria N (2019) Amitriptyline at an environmentally relevant concentration alters the profile of metabolites beyond monoamines in gilt-head bream. Environ Toxicol Chem 38(5):965–977. 10.1002/etc.4381 30702171

