# Supplementary material for: Executive functioning in wild guppies: investigating the impact of a pharmaceutical pollutant
Source: Anim Cogn. 2026 Jun 16;29(1):66. doi: 10.1007/s10071-026-02078-w (PMC13590453; doi:10.1007/s10071-026-02078-w)
Supplement: Supplementary file 1 [file 10071_2026_2078_MOESM1_ESM.docx]

**Executive functioning in wild guppies: investigating the impact of a pharmaceutical pollutant**

Eleanor R. Moore ^1,^*, Jack L. Manera ^1^, Jack A. Brand ^2,3^, Michael G. Bertram ^1,2,4,†^, Bob B.M. Wong ^1,†^

^1^ *School of Biological Sciences, Monash University, Melbourne 3800, Australia*

^2^ *Department of Wildlife, Fish and Environmental Studies, Swedish University of Agricultural Sciences, Umeå 901 83, Sweden*

^3^ *Institute of Zoology, Zoological Society of London, London NW1 4RY, United Kingdom*

^4^ *Department of Zoology, Stockholm University, Stockholm 114 18, Sweden*

* Corresponding author: Eleanor R. Moore, eleanor.moore@monash.edu

† Co-senior authors

**The following supplementary material is available for this article:**

**Supplementary material and methods**

Amitriptyline analytical protocol

**Supplementary results**

Amitriptyline exposure and fish activity

Figure S1. Modelled relationship between the mean distance fish travelled (cm) during 1-h of FMP y-maze exploration, and the total number of turns made during this time, after exposure to different amitriptyline treatments.

Figure S2. Strategy comparison of tetragram usage in fish from the control treatment during 1-h of FMP y-maze exploration.

Figure S3. Global activity (counts of all tetragrams) in fish exposed to three amitriptyline treatments during 1 h of FMP Y-maze exploration.

**Supplementary tables**

Table S1. Activity model coefficients

Table S2. Estimated trends between turns made and distance travelled

Table S3. Contrasts of trends between turns made and distance travelled

Table S4. Tetragram count model coefficients

Table S5. Marginal estimates of strategy use (counts)

Table S6. Contrasts of strategy use

Table S7. Alternation proportion model coefficients

Table S8. Marginal estimates of alternation proportions

Table S9. Contrasts of alternation proportions

Table S10. Tetragram count over time model coefficients

Table S11. Marginal estimates of alternation use over time

Table S12. Marginal estimates of other tetragram use over time

Table S13. Estimated rates of strategy adaptation over time

Table S14. Contrasts of adaptation rate over time, within treatment, by strategy

Table S15. Contrasts of adaptation rate over time, within strategy, by treatment

Table S16. Estimated rate of change in activity over time

Table S17. Contrasts of activity change rates

Table S18. Morphological measurements summary

**Supplementary materials and methods**

*Amitriptyline analytical protocol*

A 4mL aliquot of each water sample was transferred into a 4 mL vial. The following were then added to each vial, as well as to all calibration standard:

- 20 µL of 90 ng/mL amitriptyline-D3 (Limpo, USA) prepared in LC-MS/MS grade methanol (≥99.9%), which acted as an internal standard
- 20 µL of 1 µg/mL 17α-trenbolone (17α-hydroxyestra-4,9,11-trien-3-one; CAS: 80657-17-6; Novachem, Germany) prepared in LC-MS/MS grade methanol (≥99.9%), which acted as a surrogate standard

All samples were filtered using RC syringeless filter vials (0.2 µm) and analysed using LC-MS/MS. A 40 µL injection was performed in electrospray ionisation (ESI) mode onto a Biphenyl column (2.7 µm, 100 mm × 2.1 mm). For each analyte, two transitions were monitored:

- Amitriptyline: quantification ion 278.20 → 105.20 (collision energy −24 V); confirmatory ion 287.20 → 233.20 (−18 V).
- 17α-trenbolone surrogate standard: quantification ion 271.20 → 253.10 (−22 V); confirmatory ion 271.20 → 107.10 (−34 V).

The mobile phase gradient began with an initial concentration of 95% of 0.1% formic acid in water and 5% methanol (0–0.5 min). This was decreased slowly to 2% of 0.1% formic acid in water (0.5–8.75 min) and held at this concentration for a further 0.25 min (8.75–9 min), before being returned to 95% of 0.1% formic acid in water (9–14.1 min).

For calibration, an eight-point curve was used. An intermediate solution of amitriptyline (0.1 µg/mL in LC-MS/MS grade methanol) was prepared and diluted to generate a water spike at 0.001 µg/mL. The calibration standards were processed identically and alongside the experimental samples. The method achieved a limit of quantification (LOQ) of 5 ng/L for amitriptyline. No amitriptyline contamination was detected in any of the control tank samples.

**Supplementary results**

*Amitriptyline exposure and fish activity*

Regardless of amitriptyline treatment, fish that made more turns during the trial covered a greater distance (Figure S1; Table S2). Stereotypic behaviour in this task may be characterised by high activity in one arm of the maze without making many turns, which would indicate a departure from normal exploration. The presence of more individuals performing this behaviour in any one treatment group would deform the slope of this relationship, as these fish would demonstrate higher than average distance travelled for the total number of turns they completed. However, the fact that none of these treatment group trends differ meaningfully from one another indicates that any cases where fish may exhibit such stereotypic behaviour are evenly distributed across treatments (Table S3). This also provides validation for the use of total turns completed as a proxy for activity in the rest of the analysis.

Within all treatment groups, general activity decreased throughout the trial (Figure S3). When the number of tetragrams performed in each 10-min bin was averaged regardless of strategy, the rate at which fish turned decreased with every successive 10-minute time bin (Table S16). However, these trends showed negligible differences between treatment groups (Table S17).

**Fig. S1** Modelled relationship between the mean distance fish travelled during 1-h of FMP y-maze exploration (cm), and the total number of turns made during this time (range; 8, 406), after exposure to different amitriptyline treatments: control (0 ng/L, *n* = 47; blue), low amitriptyline (9 ng/L, *n* = 50; yellow), or high amitriptyline (309 ng/L, *n* = 45; red). The shaded regions represent 95% credible intervals.

**Fig. S2** Strategy comparison of tetragram usage in fish from the control treatment (0 ng/L amitriptyline, *n* = 47) during 1-h of FMP y-maze exploration. The mean count of alternation tetragrams performed (i.e. LRLR and RLRL; green) is compared to the mean count of the remaining 14 other tetragrams (grey). The points represent the model-estimated marginal means, error bars represent the 95% credible intervals and density plots represent the posterior distribution.

**Fig. S3** Global activity (counts of all tetragrams) in fish exposed to three amitriptyline treatments: control (0 ng/L, *n* = 47; blue), low amitriptyline (9 ng/L, *n* = 50; yellow), or high amitriptyline (309 ng/L, *n* = 45; red), during 1 h of FMP Y-maze exploration. The mean number of all tetragrams that fish performed is displayed for each 10-min time bin of the trial, for each treatment group. The shaded regions represent 95% credible intervals.

**Supplementary tables**

**Table S1:** Posterior summaries of fixed effects from the activity model. Shown are parameter estimates, standard errors, and 95% credible intervals.

| Parameter | Estimate | Est. error | Lower CrI | Upper CrI |
| --- | --- | --- | --- | --- |
| Intercept | −1.672 | 0.238 | −2.137 | −1.205 |
| total_turns | 0.010 | 0.001 | 0.008 | 0.013 |
| treatmenth | −0.150 | 0.333 | −0.798 | 0.500 |
| treatmentl | −0.209 | 0.319 | −0.841 | 0.409 |
| sexM | 0.073 | 0.127 | −0.182 | 0.320 |
| round2 | 0.128 | 0.157 | −0.179 | 0.424 |
| round3 | 0.074 | 0.148 | −0.219 | 0.365 |
| round4 | 0.073 | 0.162 | −0.243 | 0.394 |
| scaled_std_length | 0.055 | 0.061 | −0.174 | 0.065 |
| total_turns:treatmenth | 0.000 | 0.002 | −0.003 | 0.004 |
| total_turns:treatmentl | −0.000 | 0.002 | −0.004 | 0.004 |

**Table S2:** Posterior estimates of trends between the total number of turns made and the total distance travelled, for each amitriptyline treatment, in 1 h of exploration in the FMP Y-maze, with 95% credible intervals.

| **Treatment** | **Estimated marginal trend** | **Lower CrI** | **Upper CrI** |
| --- | --- | --- | --- |
| Control | 8.919 | 6.490 | 11.275 |
| Low | 8.834 | 6.808 | 10.832 |
| High | 9.137 | 6.790 | 11.440 |

**Table S3:** Pairwise contrasts of trends between total turns made and total distance travelled, in 1 h of FMP Y-maze exploration, between amitriptyline treatment groups, with 95% credible intervals.

| **Contrast** | **Estimated marginal difference in trend** | **Lower CrI** | **Upper CrI** |
| --- | --- | --- | --- |
| High – Control | 0.237 | −2.846 | 3.449 |
| Low – Control | −0.047 | −3.127 | 2.961 |
| Low – High | −0.285 | −3.319 | 2.866 |

**Table S4:** Posterior summaries of fixed effects from the whole-trial tetragram count model. Shown are parameter estimates, standard errors, and 95% credible intervals.

| Parameter | Estimate | Est. error | Lower CrI | Upper CrI |
| --- | --- | --- | --- | --- |
| Intercept | 2.008 | 0.081 | 1.847 | 2.163 |
| tetragramLLLR | −0.060 | 0.094 | −0.242 | 0.127 |
| tetragramLLRL | 0.051 | 0.092 | −0.131 | 0.231 |
| tetragramLLRR | −0.110 | 0.095 | −0.293 | 0.076 |
| tetragramLRLL | 0.075 | 0.093 | −0.105 | 0.256 |
| tetragramLRLR | 0.512 | 0.087 | 0.342 | 0.685 |
| tetragramLRRL | 0.027 | 0.094 | −0.153 | 0.213 |
| tetragramLRRR | −0.163 | 0.095 | −0.351 | 0.024 |
| tetragramRLLL | −0.064 | 0.095 | −0.248 | 0.123 |
| tetragramRLLR | 0.008 | 0.094 | −0.176 | 0.193 |
| tetragramRLRL | 0.539 | 0.087 | 0.372 | 0.710 |
| tetragramRLRR | −0.029 | 0.093 | −0.209 | 0.153 |
| tetragramRRLL | −0.145 | 0.095 | −0.329 | 0.042 |
| tetragramRRLR | 0.002 | 0.094 | −0.184 | 0.188 |
| tetragramRRRL | −0.170 | 0.095 | −0.357 | 0.017 |
| tetragramRRRR | −0.147 | 0.096 | −0.332 | 0.042 |
| treatmenth | 0.062 | 0.101 | −0.133 | 0.263 |
| treatmentl | −0.019 | 0.099 | −0.210 | 0.176 |
| scaled_total_turns | 0.356 | 0.041 | 0.277 | 0.435 |
| sexM | −0.003 | 0.040 | −0.081 | 0.075 |
| round2 | 0.172 | 0.047 | 0.080 | 0.264 |
| round3 | 0.161 | 0.046 | 0.070 | 0.251 |
| round4 | 0.133 | 0.050 | 0.035 | 0.231 |
| scaled_standard_length | −0.046 | 0.019 | −0.084 | −0.008 |
| tetragramLLLR:treatmenth | −0.072 | 0.133 | −0.333 | 0.187 |
| tetragramLLRL:treatmenth | 0.009 | 0.129 | −0.251 | 0.259 |
| tetragramLLRR:treatmenth | −0.078 | 0.133 | −0.338 | 0.184 |
| tetragramLRLL:treatmenth | −0.033 | 0.131 | −0.292 | 0.220 |
| tetragramLRLR:treatmenth | −0.075 | 0.123 | −0.316 | 0.163 |
| tetragramLRRL:treatmenth | −0.027 | 0.131 | −0.287 | 0.231 |
| tetragramLRRR:treatmenth | −0.052 | 0.134 | −0.315 | 0.208 |
| tetragramRLLL:treatmenth | −0.068 | 0.134 | −0.332 | 0.192 |
| tetragramRLLR:treatmenth | 0.004 | 0.132 | −0.252 | 0.259 |
| tetragramRLRL:treatmenth | −0.111 | 0.123 | −0.350 | 0.132 |
| tetragramRLRR:treatmenth | −0.006 | 0.133 | −0.263 | 0.250 |
| tetragramRRLL:treatmenth | −0.04 | 0.133 | −0.300 | 0.217 |
| tetragramRRLR:treatmenth | −0.048 | 0.132 | −0.311 | 0.208 |
| tetragramRRRL:treatmenth | −0.057 | 0.134 | −0.321 | 0.205 |
| tetragramRRRR:treatmenth | −0.025 | 0.134 | −0.287 | 0.238 |
| tetragramLLLR:treatmentl | −0.058 | 0.131 | −0.317 | 0.201 |
| tetragramLLRL:treatmentl | −0.063 | 0.129 | −0.314 | 0.189 |
| tetragramLLRR:treatmentl | 0.024 | 0.131 | −0.234 | 0.278 |
| tetragramLRLL:treatmentl | −0.061 | 0.129 | −0.311 | 0.187 |
| tetragramLRLR:treatmentl | −0.019 | 0.122 | −0.259 | 0.219 |
| tetragramLRRL:treatmentl | −0.029 | 0.130 | −0.286 | 0.227 |
| tetragramLRRR:treatmentl | 0.084 | 0.131 | −0.173 | 0.340 |
| tetragramRLLL:treatmentl | −0.036 | 0.131 | −0.286 | 0.220 |
| tetragramRLLR:treatmentl | 0.010 | 0.131 | −0.249 | 0.265 |
| tetragramRLRL:treatmentl | −0.027 | 0.122 | −0.266 | 0.211 |
| tetragramRLRR:treatmentl | 0.014 | 0.129 | −0.238 | 0.265 |
| tetragramRRLL:treatmentl | 0.054 | 0.132 | −0.205 | 0.313 |
| tetragramRRLR:treatmentl | 0.011 | 0.129 | −0.241 | 0.265 |
| tetragramRRRL:treatmentl | 0.096 | 0.131 | −0.162 | 0.351 |
| tetragramRRRR:treatmentl | 0.090 | 0.131 | −0.17 | 0.346 |
| tetragramLLLR:scaled_total_turns | 0.030 | 0.054 | −0.075 | 0.138 |
| tetragramLLRL:scaled_total_turns | 0.065 | 0.053 | −0.038 | 0.171 |
| tetragramLLRR:scaled_total_turns | 0.024 | 0.055 | −0.082 | 0.132 |
| tetragramLRLL:scaled_total_turns | 0.058 | 0.052 | −0.042 | 0.161 |
| tetragramLRLR:scaled_total_turns | 0.299 | 0.051 | 0.198 | 0.397 |
| tetragramLRRL:scaled_total_turns | 0.104 | 0.053 | −0.002 | 0.208 |
| tetragramLRRR:scaled_total_turns | 0.054 | 0.054 | −0.051 | 0.163 |
| tetragramRLLL:scaled_total_turns | 0.021 | 0.055 | −0.087 | 0.129 |
| tetragramRLLR:scaled_total_turns | 0.063 | 0.053 | −0.041 | 0.166 |
| tetragramRLRL:scaled_total_turns | 0.287 | 0.050 | 0.191 | 0.385 |
| tetragramRLRR:scaled_total_turns | 0.136 | 0.054 | 0.032 | 0.244 |
| tetragramRRLL:scaled_total_turns | 0.029 | 0.055 | −0.077 | 0.138 |
| tetragramRRLR:scaled_total_turns | 0.124 | 0.054 | 0.020 | 0.232 |
| tetragramRRRL:scaled_total_turns | 0.053 | 0.054 | −0.053 | 0.161 |
| tetragramRRRR:scaled_total_turns | 0.088 | 0.055 | −0.017 | 0.197 |

**Table S5:** Posterior marginal mean estimates of the frequency of each strategy across 1 h of FMP Y-maze exploration, by treatment, with 95% credible intervals. LRLR and RLRL tetragrams have been grouped as ‘Alternations’ and all other tetragrams have been grouped as ‘Others’.

| **Treatment** | **Strategy** | **Estimate** | **Lower CrI** | **Upper CrI** |
| --- | --- | --- | --- | --- |
| Control | Alternations | 14.193 | 12.812 | 15.553 |
|  | Others | 7.998 | 7.460 | 8.485 |
| Low | Alternations | 13.614 | 12.289 | 14.876 |
|  | Others | 7.908 | 7.409 | 8.410 |
| High | Alternations | 13.764 | 12.443 | 15.167 |
|  | Others | 8.229 | 7.702 | 8.762 |

**Table S6:** Pairwise contrasts of the frequency of each strategy across 1 h of FMP Y-maze exploration, by treatment, with 95% credible intervals. LRLR and RLRL tetragrams have been grouped as ‘Alternations’ and all other tetragrams have been grouped as ‘Others’.

| **Treatment** | **Contrast** | **Estimated difference** | **Lower CrI** | **Upper CrI** |
| --- | --- | --- | --- | --- |
| Control | Alternations –Others | 6.195 | 5.019 | 7.457 |
| Low | Alternations –Others | 5.705 | 4.606 | 6.901 |
| High | Alternations –Others | 5.535 | 4.377 | 6.802 |

**Table S7:** Posterior summaries of fixed effects from the alternation proportion model. Shown are parameter estimates, standard errors, and 95% credible intervals.

| Parameter | Estimate | Est. error | Lower CrI | Upper CrI |
| --- | --- | --- | --- | --- |
| Intercept | −1.535 | 0.135 | −1.805 | −1.272 |
| treatmenth | 0.079 | 0.147 | −0.211 | 0.364 |
| treatmentl | 0.066 | 0.146 | −0.226 | 0.353 |
| scaled_total_turns | 0.279 | 0.101 | 0.076 | 0.475 |
| sexM | 0.113 | 0.135 | −0.143 | 0.386 |
| scaled_standard_length | −0.054 | 0.059 | −0.171 | 0.059 |
| treatmenth:scaled_total_turns | −0.140 | 0.138 | −0.404 | 0.131 |
| treatmentl:scaled_total_turns | −0.003 | 0.134 | −0.261 | 0.264 |

**Table S8:** Posterior marginal mean estimates of the proportion of tetragrams that were alternations across 1 h of FMP Y-maze exploration, by treatment, with 95% credible intervals.

| **Treatment** | **Estimate** | **Lower CrI** | **Upper CrI** |
| --- | --- | --- | --- |
| Control | 0.186 | 0.156 | 0.218 |
| Low | 0.196 | 0.165 | 0.229 |
| High | 0.198 | 0.167 | 0.232 |

**Table S9:** Pairwise contrasts of the proportion of tetragrams that were alternations across 1 h of FMP Y-maze exploration, by treatment, with 95% credible intervals.

| **Contrast** | **Estimated difference** | **Lower CrI** | **Upper CrI** |
| --- | --- | --- | --- |
| High – Control | 0.012 | −0.033 | 0.056 |
| Low – Control | 0.010 | −0.036 | 0.053 |
| High – Low | 0.002 | −0.042 | 0.048 |

**Table S10:** Posterior summaries of fixed effects from the time-binned tetragram count model. Shown are parameter estimates, standard errors, and 95% credible intervals.

| Parameter | Estimate | Est. error | Lower CrI | Upper CrI |
| --- | --- | --- | --- | --- |
| Intercept | 0.233 | 0.112 | 0.008 | 0.446 |
| tetragramLLLR | 0.023 | 0.148 | −0.269 | 0.314 |
| tetragramLLRL | 0.274 | 0.142 | −0.004 | 0.555 |
| tetragramLLRR | −0.197 | 0.156 | −0.502 | 0.102 |
| tetragramLRLL | 0.256 | 0.141 | −0.019 | 0.539 |
| tetragramLRLR | 0.707 | 0.129 | 0.459 | 0.969 |
| tetragramLRRL | −0.005 | 0.149 | −0.291 | 0.291 |
| tetragramLRRR | −0.255 | 0.155 | −0.555 | 0.055 |
| tetragramRLLL | 0.004 | 0.149 | −0.288 | 0.299 |
| tetragramRLLR | 0.099 | 0.145 | −0.182 | 0.386 |
| tetragramRLRL | 0.704 | 0.128 | 0.459 | 0.959 |
| tetragramRLRR | −0.063 | 0.147 | −0.347 | 0.230 |
| tetragramRRLL | −0.226 | 0.154 | −0.526 | 0.083 |
| tetragramRRLR | −0.065 | 0.147 | −0.35 | 0.225 |
| tetragramRRRL | −0.219 | 0.155 | −0.521 | 0.091 |
| tetragramRRRR | −0.199 | 0.156 | −0.504 | 0.103 |
| bin | −0.023 | 0.030 | −0.08 | 0.037 |
| treatmenth | 0.026 | 0.151 | −0.268 | 0.325 |
| treatmentl | −0.21 | 0.157 | −0.508 | 0.107 |
| scaled_total_turns | 0.524 | 0.010 | 0.504 | 0.543 |
| sexM | 0.025 | 0.036 | −0.045 | 0.097 |
| round2 | 0.175 | 0.044 | 0.091 | 0.265 |
| round3 | 0.122 | 0.044 | 0.038 | 0.210 |
| round4 | 0.125 | 0.046 | 0.036 | 0.216 |
| scaled_standard_length | −0.047 | 0.018 | −0.082 | −0.014 |
| tetragramLLLR:bin | −0.038 | 0.043 | −0.124 | 0.046 |
| tetragramLLRL:bin | −0.082 | 0.042 | −0.166 | −0.001 |
| tetragramLLRR:bin | 0.017 | 0.044 | −0.069 | 0.102 |
| tetragramLRLL:bin | −0.069 | 0.042 | −0.152 | 0.011 |
| tetragramLRLR:bin | −0.043 | 0.037 | −0.118 | 0.030 |
| tetragramLRRL:bin | 0.006 | 0.042 | −0.078 | 0.088 |
| tetragramLRRR:bin | 0.026 | 0.043 | −0.061 | 0.110 |
| tetragramRLLL:bin | −0.033 | 0.043 | −0.119 | 0.050 |
| tetragramRLLR:bin | −0.042 | 0.042 | −0.125 | 0.038 |
| tetragramRLRL:bin | −0.041 | 0.037 | −0.113 | 0.030 |
| tetragramRLRR:bin | 0.016 | 0.042 | −0.066 | 0.097 |
| tetragramRRLL:bin | 0.016 | 0.044 | −0.069 | 0.102 |
| tetragramRRLR:bin | 0.020 | 0.042 | −0.064 | 0.102 |
| tetragramRRRL:bin | 0.010 | 0.044 | −0.078 | 0.094 |
| tetragramRRRR:bin | 0.039 | 0.044 | −0.046 | 0.125 |
| tetragramLLLR:treatmenth | −0.250 | 0.210 | −0.657 | 0.166 |
| tetragramLLRL:treatmenth | −0.201 | 0.200 | −0.592 | 0.192 |
| tetragramLLRR:treatmenth | −0.139 | 0.220 | −0.578 | 0.291 |
| tetragramLRLL:treatmenth | −0.223 | 0.201 | −0.617 | 0.170 |
| tetragramLRLR:treatmenth | 0.099 | 0.181 | −0.258 | 0.451 |
| tetragramLRRL:treatmenth | 0.032 | 0.207 | −0.375 | 0.437 |
| tetragramLRRR:treatmenth | 0.081 | 0.218 | −0.345 | 0.506 |
| tetragramRLLL:treatmenth | −0.208 | 0.210 | −0.623 | 0.207 |
| tetragramRLLR:treatmenth | −0.073 | 0.203 | −0.479 | 0.320 |
| tetragramRLRL:treatmenth | 0.061 | 0.180 | −0.295 | 0.405 |
| tetragramRLRR:treatmenth | 0.232 | 0.203 | −0.175 | 0.624 |
| tetragramRRLL:treatmenth | 0.015 | 0.217 | −0.411 | 0.444 |
| tetragramRRLR:treatmenth | 0.133 | 0.205 | −0.273 | 0.537 |
| tetragramRRRL:treatmenth | 0.050 | 0.217 | −0.377 | 0.472 |
| tetragramRRRR:treatmenth | 0.210 | 0.220 | −0.219 | 0.648 |
| tetragramLLLR:treatmentl | 0.044 | 0.215 | −0.386 | 0.455 |
| tetragramLLRL:treatmentl | −0.093 | 0.207 | −0.507 | 0.305 |
| tetragramLLRR:treatmentl | 0.250 | 0.223 | −0.201 | 0.683 |
| tetragramLRLL:treatmentl | −0.119 | 0.204 | −0.526 | 0.271 |
| tetragramLRLR:treatmentl | 0.232 | 0.185 | −0.139 | 0.583 |
| tetragramLRRL:treatmentl | 0.218 | 0.211 | −0.198 | 0.622 |
| tetragramLRRR:treatmentl | 0.338 | 0.217 | −0.092 | 0.754 |
| tetragramRLLL:treatmentl | 0.048 | 0.214 | −0.372 | 0.468 |
| tetragramRLLR:treatmentl | 0.060 | 0.208 | −0.355 | 0.459 |
| tetragramRLRL:treatmentl | 0.230 | 0.185 | −0.138 | 0.585 |
| tetragramRLRR:treatmentl | 0.259 | 0.207 | −0.159 | 0.653 |
| tetragramRRLL:treatmentl | 0.306 | 0.214 | −0.120 | 0.721 |
| tetragramRRLR:treatmentl | 0.220 | 0.209 | −0.190 | 0.620 |
| tetragramRRRL:treatmentl | 0.273 | 0.217 | −0.163 | 0.695 |
| tetragramRRRR:treatmentl | 0.409 | 0.223 | −0.027 | 0.842 |
| bin:treatmenth | 0.015 | 0.041 | −0.065 | 0.096 |
| bin:treatmentl | 0.056 | 0.042 | −0.029 | 0.137 |
| tetragramLLLR:bin:treatmenth | 0.060 | 0.060 | −0.059 | 0.176 |
| tetragramLLRL:bin:treatmenth | 0.072 | 0.057 | −0.042 | 0.186 |
| tetragramLLRR:bin:treatmenth | 0.021 | 0.061 | −0.100 | 0.140 |
| tetragramLRLL:bin:treatmenth | 0.068 | 0.057 | −0.045 | 0.180 |
| tetragramLRLR:bin:treatmenth | −0.046 | 0.052 | −0.149 | 0.056 |
| tetragramLRRL:bin:treatmenth | −0.014 | 0.058 | −0.130 | 0.099 |
| tetragramLRRR:bin:treatmenth | −0.042 | 0.061 | −0.162 | 0.075 |
| tetragramRLLL:bin:treatmenth | 0.047 | 0.060 | −0.071 | 0.166 |
| tetragramRLLR:bin:treatmenth | 0.034 | 0.058 | −0.077 | 0.146 |
| tetragramRLRL:bin:treatmenth | −0.038 | 0.051 | −0.138 | 0.062 |
| tetragramRLRR:bin:treatmenth | −0.075 | 0.058 | −0.189 | 0.041 |
| tetragramRRLL:bin:treatmenth | −0.014 | 0.061 | −0.132 | 0.105 |
| tetragramRRLR:bin:treatmenth | −0.052 | 0.059 | −0.167 | 0.061 |
| tetragramRRRL:bin:treatmenth | −0.032 | 0.061 | −0.152 | 0.089 |
| tetragramRRRR:bin:treatmenth | −0.076 | 0.061 | −0.201 | 0.042 |
| tetragramLLLR:bin:treatmentl | −0.021 | 0.061 | −0.138 | 0.099 |
| tetragramLLRL:bin:treatmentl | 0.017 | 0.059 | −0.098 | 0.135 |
| tetragramLLRR:bin:treatmentl | −0.062 | 0.061 | −0.182 | 0.059 |
| tetragramLRLL:bin:treatmentl | 0.025 | 0.058 | −0.086 | 0.141 |
| tetragramLRLR:bin:treatmentl | −0.048 | 0.052 | −0.147 | 0.057 |
| tetragramLRRL:bin:treatmentl | −0.066 | 0.059 | −0.181 | 0.051 |
| tetragramLRRR:bin:treatmentl | −0.083 | 0.061 | −0.200 | 0.037 |
| tetragramRLLL:bin:treatmentl | −0.022 | 0.061 | −0.140 | 0.099 |
| tetragramRLLR:bin:treatmentl | −0.007 | 0.058 | −0.118 | 0.111 |
| tetragramRLRL:bin:treatmentl | −0.039 | 0.052 | −0.141 | 0.065 |
| tetragramRLRR:bin:treatmentl | −0.078 | 0.058 | −0.190 | 0.038 |
| tetragramRRLL:bin:treatmentl | −0.075 | 0.060 | −0.191 | 0.045 |
| tetragramRRLR:bin:treatmentl | −0.061 | 0.058 | −0.175 | 0.055 |
| tetragramRRRL:bin:treatmentl | −0.056 | 0.060 | −0.172 | 0.065 |
| tetragramRRRR:bin:treatmentl | −0.116 | 0.062 | −0.236 | 0.009 |

**Table S11:** Posterior marginal mean estimates of the number of alternations performed in each 10-minute time bin, by treatment, with 95% credible intervals.

| Treatment | Time bin | Estimate | Lower CrI | Upper CrI |
| --- | --- | --- | --- | --- |
| Control | 1 | 2.646 | 2.384 | 2.892 |
|  | 2 | 2.479 | 2.282 | 2.675 |
|  | 3 | 2.324 | 2.161 | 2.502 |
|  | 4 | 2.179 | 2.009 | 2.360 |
|  | 5 | 2.044 | 1.853 | 2.257 |
|  | 6 | 1.918 | 1.681 | 2.150 |
| Low | 1 | 2.736 | 2.482 | 2.990 |
|  | 2 | 2.596 | 2.406 | 2.804 |
|  | 3 | 2.463 | 2.298 | 2.646 |
|  | 4 | 2.338 | 2.164 | 2.520 |
|  | 5 | 2.221 | 2.025 | 2.429 |
|  | 6 | 2.109 | 1.888 | 2.357 |
| High | 1 | 2.860 | 2.594 | 3.130 |
|  | 2 | 2.609 | 2.406 | 2.811 |
|  | 3 | 2.380 | 2.218 | 2.560 |
|  | 4 | 2.176 | 1.998 | 2.339 |
|  | 5 | 1.984 | 1.797 | 2.171 |
|  | 6 | 1.812 | 1.611 | 2.037 |

**Table S12:** Posterior marginal mean estimates of the number of other tetragrams performed in each 10-minute time bin, by treatment, with 95% credible intervals.

| Treatment | Time bin | Estimate | Lower CrI | Upper CrI |
| --- | --- | --- | --- | --- |
| Control | 1 | 1.312 | 1.225 | 1.406 |
|  | 2 | 1.266 | 1.192 | 1.346 |
|  | 3 | 1.224 | 1.154 | 1.295 |
|  | 4 | 1.186 | 1.114 | 1.257 |
|  | 5 | 1.152 | 1.077 | 1.230 |
|  | 6 | 1.122 | 1.041 | 1.213 |
| Low | 1 | 1.253 | 1.164 | 1.335 |
|  | 2 | 1.228 | 1.153 | 1.305 |
|  | 3 | 1.206 | 1.135 | 1.278 |
|  | 4 | 1.185 | 1.115 | 1.259 |
|  | 5 | 1.165 | 1.088 | 1.241 |
|  | 6 | 1.148 | 1.063 | 1.234 |
| High | 1 | 1.330 | 1.242 | 1.423 |
|  | 2 | 1.306 | 1.228 | 1.387 |
|  | 3 | 1.283 | 1.212 | 1.360 |
|  | 4 | 1.262 | 1.191 | 1.340 |
|  | 5 | 1.243 | 1.166 | 1.327 |
|  | 6 | 1.226 | 1.137 | 1.317 |

**Table S13:** Posterior estimates of adaptation rates (change in frequency across 10-minute time bins) by strategy and treatment, with 95% credible intervals. LRLR and RLRL tetragrams have been grouped as ‘Alternations’ and all other tetragrams have been grouped as ‘Others’.

| Strategy | Treatment | Trend over time bins | Lower CrI | Upper CrI |
| --- | --- | --- | --- | --- |
| Alternations | Control | −0.145 | −0.215 | −0.075 |
|  | Low | −0.125 | −0.191 | −0.054 |
|  | High | −0.208 | −0.279 | −0.141 |
| Others | Control | −0.037 | −0.057 | −0.016 |
|  | Low | −0.021 | −0.040 | −0.002 |
|  | High | −0.021 | −0.041 | −0.001 |

**Table S14:** Pairwise contrasts of adaptation rates (change in frequency across 10-minute time bins), between strategies, for each treatment, with 95% credible intervals. LRLR and RLRL tetragrams have been grouped as ‘Alternations’ and all other tetragrams have been grouped as ‘Others’.

| Treatment | Contrast | Estimated difference in trend | Lower CrI | Upper CrI |
| --- | --- | --- | --- | --- |
| Control | Alternations – Other | −0.107 | −0.180 | −0.036 |
| Low | Alternations – Other | −0.104 | −0.176 | −0.035 |
| High | Alternations – Other | −0.187 | −0.257 | −0.116 |

**Table S15:** Pairwise contrasts of adaptation rates (change in frequency across 10-minute time bins) for each strategy, between treatments, with 95% credible intervals. LRLR and RLRL tetragrams have been grouped as ‘Alternations’ and all other tetragrams have been grouped as ‘Others’.

| Strategy | Contrast | Estimated difference in trend | Lower CrI | Upper CrI |
| --- | --- | --- | --- | --- |
| Alternations | High – Control | −0.063 | −0.161 | 0.034 |
|  | Low – Control | 0.020 | −0.074 | 0.120 |
|  | High – Low | −0.083 | −0.181 | 0.011 |
| Others | High – Control | 0.017 | −0.011 | 0.045 |
|  | Low – Control | 0.017 | −0.011 | 0.043 |
|  | High – Low | 0.000 | −0.027 | 0.027 |

**Table S16:** Posterior estimates of activity (rate at which tetragrams are performed across 10-minute time bins) by treatment, with 95% credible intervals.

| Treatment | Trend over time bins | Lower CrI | Upper CrI |
| --- | --- | --- | --- |
| Control | −0.051 | −0.072 | −0.031 |
| Low | −0.034 | −0.052 | −0.014 |
| High | −0.044 | −0.065 | −0.025 |

**Table S17:** Pairwise contrasts of activity (rate at which tetragrams are performed across 10-minute time bins), by treatment, with 95% credible intervals.

| Contrast | Estimated difference in trend | Lower CrI | Upper CrI |
| --- | --- | --- | --- |
| High – Control | 0.007 | –0.021 | 0.034 |
| Low – Control | 0.017 | –0.010 | 0.044 |
| High – Low | –0.010 | –0.037 | 0.016 |

**Table S18:** Morphological information for all individuals that participated in the FMP Y-maze, by treatment.

| Treatment | Number of fish | Standard length ± SD (cm) |
| --- | --- | --- |
| Control | 47 | 15.95 ± 1.79 |
| Low | 50 | 16.64 ± 3.22 |
| High | 45 | 16.61 ± 2.98 |
